# Empirical Comparison of Diffusion Kurtosis Imaging and Diffusion Basis Spectrum Imaging Using the Same Acquisition in Healthy Young Adults

**DOI:** 10.3389/fneur.2017.00118

**Published:** 2017-03-29

**Authors:** Sijia Wang, Daniel J. Peterson, Yong Wang, Qing Wang, Thomas J. Grabowski, Wenbin Li, Tara M. Madhyastha

**Affiliations:** ^1^Institute of Diagnostic and Interventional Radiology, Shanghai Jiao Tong University Affiliated Sixth People’s Hospital, Shanghai, China; ^2^Department of Radiology, University of Washington, Seattle, WA, USA; ^3^Department of Obstetrics and Gynecology, Washington University, St. Louis, MO, USA; ^4^Department of Radiology, Washington University, St. Louis, MO, USA; ^5^Department of Biomedical Engineering, Washington University, St. Louis, MO, USA; ^6^Department of Neurology, University of Washington, Seattle, WA, USA

**Keywords:** diffusion basis spectrum imaging, diffusion kurtosis imaging, diffusion tensor imaging, model comparison, diffusion magnetic resonance imaging

## Abstract

As diffusion tensor imaging gains widespread use, many researchers have been motivated to go beyond the tensor model and fit more complex diffusion models, to gain a more complete description of white matter microstructure and associated pathology. Two such models are diffusion kurtosis imaging (DKI) and diffusion basis spectrum imaging (DBSI). It is not clear which DKI parameters are most closely related to DBSI parameters, so in the interest of enabling comparisons between DKI and DBSI studies, we conducted an empirical survey of the interrelation of these models in 12 healthy volunteers using the same diffusion acquisition. We found that mean kurtosis is positively associated with the DBSI fiber ratio and negatively associated with the hindered ratio. This was primarily driven by the radial component of kurtosis. The axial component of kurtosis was strongly and specifically correlated with the restricted ratio. The joint spatial distributions of DBSI and DKI parameters are tissue-dependent and stable across healthy individuals. Our contribution is a better understanding of the biological interpretability of the parameters generated by the two models in healthy individuals.

## Introduction

Diffusion magnetic resonance imaging (MRI) is an important technique for measuring white matter (WM) microstructure *in vivo*. There are a variety of techniques to model diffusion MRI data, with the goal of non-invasively deriving quantities that reflect the normal or pathological state of the tissue.

Diffusion tensor imaging (DTI) is a classic diffusion MRI modeling technique that models the dispersion of water molecules assuming a Gaussian distribution, which can be visualized in three dimensions as an elliptical isosurface. The properties of the diffusion tensor can be quantified by commonly used DTI statistics, including mean diffusivity (MD), a directionally averaged measure of diffusion; axial diffusivity (AD), diffusion along the axial diffusion direction; radial diffusivity (RD), diffusion perpendicular to the axial diffusion direction; and fractional anisotropy (FA), a measure of the directionality of diffusion. Extensive studies have shown that DTI is sensitive to microstructural changes, but the measures lack specificity to reflect anatomical complexity and heterogeneous pathology. Different kinds of microstructural disruption result in similar changes to DTI parameters. For example, either the accumulation of extracellular fluid (edema) or degradation of the myelin sheath (demyelination) can lead to increased MD and RD, as well as decreased FA.

These ambiguities in interpretation of DTI-derived quantities, as well as recent advances in MRI image acquisition techniques, such as simultaneous multi-slice acquisition (1), have motivated an interest in developing more complex diffusion MRI models to better characterize the diffusion properties of WM without directly modeling biological microstructure. One of the first steps to improving the accuracy of the model is to relax the assumption that the dispersion of water molecules is Gaussian. Non-Gaussian diffusion is of biological interest, because it represents the existence of complex barriers and compartments within a voxel. Diffusion kurtosis imaging (DKI) is a commonly used approach and a focus of this paper, but other approaches exist that can also indirectly account for non-Gaussianity of the diffusion-weighted signal, such as spherical deconvolution (2, 3) and restriction spectrum imaging (4).

The DKI approach is an extension of the diffusion tensor model, where the deviations from Gaussianity are modeled as a 3 × 3 × 3 × 3 kurtosis tensor. Due to the extensive symmetry, the kurtosis tensor has only 15 independent parameters, which, when added to the original 6 parameters in DTI bring the full DKI model to 21 parameters. Rotationally invariant scalar diffusion kurtosis indices can be calculated from these parameters, analogous to the diffusion indices in DTI. Kurtosis indices that are commonly used as biomarkers are mean kurtosis (MK), axial kurtosis (AK), and radial kurtosis (RK). These represent the mean deviation from Gaussianity, the directional deviation from Gaussianity along the axial diffusion direction, and the directional deviation perpendicular to the axial diffusion direction. Recent work has shown that kurtosis indices can be more sensitive to microstructural damage than parameters from DTI models (5–7) and that the addition, and the directional deviation perpendicular to the axial diffusion direction. Recent work has shown that kurtosis indices can be more sensitive to microstructural damage than parameters from DTI models (5–7), and that the additional parameters provide a fit to high-quality data that are both more accurate and more reliable (8). In ischemic brain injury, MK changes when water shifts from the extracellular to the intracellular space because of the failure of ion pumps (cytotoxic edema). These MK changes in edema likely result from decreased extracellular space and increased complexity of the remaining extracellular space (9).

A drawback of the DKI approach is that it does not explicitly model microstructural parameters. For this reason, multi-compartment diffusion models have been developed that model the signal as the contributions coming from multiple tissue compartments that represent different microstructural environments. Some well-known multi-compartment models are ball and stick models (10) and their successors (11), which provide information about crossing fiber, CHARMED (12), which models hindered and restricted diffusion, NODDI (13), which models neurite orientation dispersion, AxCaliber (14) and ActiveAx (10), which model axon diameter, and diffusion basis spectrum imaging (DBSI), which is the focus of this paper.

Diffusion basis spectrum imaging models the diffusion-weighted MRI signal as a linear combination of a basis set of cylindrically symmetric tensors (15) and a spectrum of isotropic tensors with apparent diffusivity covering the entire physiological range (16). With multiple tensors representing both the anisotropic axonal fibers and their surrounding environment, one can describe a greater range of microstructural environments than DTI models (16, 17). By specifically modeling the sub-voxel pathologies, DBSI can derive parameters with a more specific pathophysiological interpretation. This has been validated by histopathological studies (16, 17). For example, inflammatory cell infiltration has been associated with the increased restricted isotropic diffusion component, reflecting the microstructural barriers (nucleus and cell membranes) that are non-directional and highly restrictive to water diffusion (16). Free water, such as cerebrospinal fluid (CSF) within ventricles, is modeled by the free isotropic diffusion components in DBSI. Isotropic diffusion tensors with intermediate diffusivity (as hindered diffusion) in DBSI reflect the extracellular fluid within complex tissue. The changes in hindered diffusion have been associated with vasogenic edema and tissue loss in the setting of multiple sclerosis (MS) or other pathology (16).

An important distinction between DKI and DBSI is that DKI is a mathematical model of the diffusion profile, while DBSI is a biophysically informed model of the tissue microstructure. DKI was developed as a general extension of DTI with the purpose of modeling higher-order diffusion properties. DKI can be used to characterize the diffusion of any fluid or gas (18) within a complex environment. In contrast, the DBSI model is particularly designed to model to the diffusion of water molecules within the microenvironment of the central nervous system. In contrast to the kurtosis tensor used in DKI, DBSI models the high-order diffusion properties associated with complex microstructural changes using multiple diffusion tensors.

Although both DKI and DBSI are different approaches to improving upon the classic DTI model, these methods have not been directly compared. In this study, we examine the relationship between the parameters calculated using these models on the same set of diffusion MRI data acquired from 12 healthy young adults. We conduct a correlational analysis of DTI, DKI, and DBSI parameters to characterize systematic similarities and differences. We hypothesize that in regions of high kurtosis in healthy young adults, DBSI compartments should reflect primarily high fiber complexity and cellularity (restricted diffusion). Finally, we examine how DTI, DBSI, and DKI parameters differ within subjects in areas of known anatomical complexity.

## Materials and Methods

### Participants

For this study, we imaged 12 controls who were all young and healthy graduate medical students at Shanghai Jiao Tong University (mean age = 28.08 years, SD = 2.54; 8 females). All subjects were recruited specifically for this study and provided informed consent. The study was approved by the Shanghai Jiao Tong University Affiliated Sixth People’s Hospital review board.

### Acquisition

In this study, diffusion-weighted images were collected on a Siemens MAGNETOM Verio 3-T scanner with a 32-channel head coil. A total of 150 diffusion-weighted images were collected across 5 different shells (30 identical spherically distributed *b*-vectors each) with *b*-values: 500, 1,000, 1,500, 2,000, and 2,500 mm/s. A single non-diffusion-weighted volume was also acquired. Imaging parameters for the diffusion acquisition were as follows: 25 2-mm slices acquired with 0.6-mm slice gap and in an ascending temporal slice order, TR = 3,900 ms, TE = 109 ms, FOV = 128 mm × 128 mm, 1.79 mm × 1.79 mm in-plane resolution. Total imaging time for the diffusion acquisition was 10 min and 10 s. Note that a T1 structural image was not acquired. Acquisition parameters were selected and optimized for DKI analysis. DBSI computation was adopted to analyze the same diffusion MRI data set.

### DTI, DKI, and DBSI Processing

Motion correction was carried out using “eddy” (19) from FMRIB (Oxford Center for Functional MRI of the Brain) Software Library (FSL) version 5.0.9, using preprocessing scripts written by DP that we have made publicly available.^1^ The diffusion tensor model was fit using RESTORE (20). RESTORE is an automated outlier detection and rejection algorithm that reduces the effect of motion and subtle artifacts. This processing produces the standard DTI scalar statistics (e.g., DTI-FA, DTI-RD, DTI-MD, and DTI-AD).

For DKI, the motion-corrected diffusion data were then smoothed (21–23) with a 4-mm full width at half maximum Gaussian kernel and fit to the diffusion kurtosis tensor model using dipy v0.10^2^ (24). This process estimates DTI parameters (DKI-FA, DKI-RD, DKI-MD, and DKI-AD) and MK, AK, and RK. To reduce the effect of singularities in Carlson’s elliptic integrals, and to constrain values to a plausible biophysical range, MK, RK, and AK were constrained to be between 0 and 3 (23, 25).

The processing pipeline was implemented using GNU Make (26), and scripts and Makefiles to implement these analyses are available from the first author. Additionally, the workflow is documented in Supplementary Material.

For DBSI, no further smoothing was applied to the motion-corrected diffusion data. All datasets were analyzed by a DBSI multi-tensor model analysis package developed in-house with Matlab (MathWorks) (16, 17). DBSI first analyzes the raw diffusion MRI signal to adaptively determine the number of anisotropic fiber components. The detailed multiple tensor model is then solved by inverting a linear matrix through a regularization process to avoid over-fitting. The weighted summation of the multiple tensors in DBSI was used to characterize the heterogeneous pathologies coexisting within the same imaging voxel. This processing results in quantification of fiber ratio (FR), water ratio (WR), restricted ratio (RR), hindered ratio (HR), as well as standard DTI parameters (DBSI-FA, DBSI-RD, DBSI-MD, and DBSI-AD).

### Analysis

Analyses were performed using tools from FSL version 5.0.9 to perform image math and advanced normalization tools (2.1.0—rc3) (27) to register images to standard space. Analyses were limited to white matter (“WM” voxels) in subject-specific space unless otherwise noted. To generate a WM mask, we used fslmaths to threshold the WM tissue prior image at voxels with 50% probability of being WM. We used the Montreal Neurological Institute (MNI) 1 mm resolution brain mask to mask out non-brain regions. We registered the FA images to the 1-mm MNI FA image to create standard space maps. For subject-specific measurements, we inverted these transforms to bring the WM and brain masks into subject-specific space.

All correlations were performed using Spearman’s rho (*r*_s_). This non-parametric correlation coefficient was used to avoid bias due to distributional assumptions. Correlations were implemented using R (28). We used IBM Statistical Package for the Social Sciences (v 22.0.0.0) to conduct paired *t*-tests to compare estimates of mean FA derived from the DTI, DKI, and DBSI diffusion models. We also calculated Spearman’s rho for the FA of each pair of models across all WM voxels and subjects in subject-specific space. To compare DKI and DBSI parameters, we calculated the pairwise Spearman’s rho of MK, RK, and AK with FR, WR, RR, and HR across all WM voxels and all subjects in subject-specific space.

We created maximal ratio maps of the whole brain for visualization purposes as follows. We registered all normalized ratio maps to the standard space FA image. Then, we computed the mean of all subjects normalized ratio maps using fslmaths. We masked these mean images with the MNI brain mask and the WM mask. We identified which quantity was highest at each voxel and constructed maps for FR, HR, WR, and RR, which were non-zero only where the ratio was the maximum at each voxel.

To examine DBSI parameters in areas of moderately high kurtosis, we used fslmaths to threshold the MK parameter map at 1. This threshold has the effect of selecting primarily major WM tracts (21). We further restricted the MK mask by limiting it to WM voxels. This mask describes areas of “high kurtosis.” We constructed maximal ratio maps for visualization purposes as described above in areas of high kurtosis for HR, WR, and RR. We also plotted the relative contribution of HR, WR, RR, and FR for each subject in areas of high kurtosis as a stacked bar chart. To visualize the results, we overlaid the maximal ratio maps within areas of high kurtosis on the standard space FA maps.

To more closely examine differences between parameter estimates from different models, we manually delineated two regions of interest (ROIs) with anatomically known fiber structure (see Supplementary Material). The first ROI is deep in the genu of the corpus callosum (CC), a coherent fiber bundle. The second ROI is in the decussation of the CC and the inferior fronto-occipital fasciculus, an area of crossing fibers (29). We identified these ROIs on the DTI-FA image for each individual. The ROI size was the same for all individuals: *x* = 9 mm (5 voxels × 1.80 mm), *y* = 3.6 mm (2 voxels × 1.80 mm), and *z* = 6.5 mm (2 voxels × 3.25 mm). Figure S1 in Supplementary Material shows, for a representative subject, the location of (A) the mask in the genu of CC and (B) the mask in crossing fibers. We used these masks to calculate the mean values of DTI-FA/AD/RD, DKI-FA/AD/RD/MK/AK/RK, and DBSI-FA/AD/RD/FR/HR/WR/RR for each individual and compared these values using paired *t*-tests.

## Results

### FA Values Calculated from DTI, DBSI, and DKI Models

**Figure 1:**
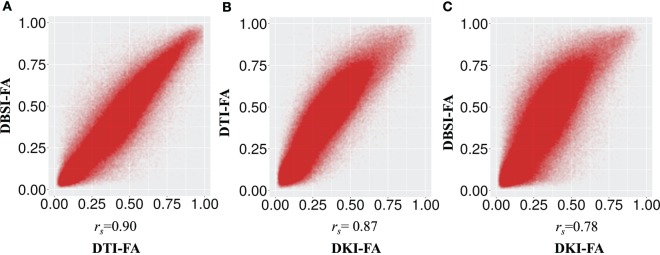
**Correlation between fractional anisotropy (FA) calculated from different models (A) diffusion tensor imaging (DTI)-FA and diffusion basis spectrum imaging (DBSI)-FA, (B) DTI-FA and smoothed diffusion kurtosis imaging (DKI)-FA, and (C) smoothed DKI-FA and DBSI-FA across all individuals and white matter voxels**. All *p* values are significant and <0.001.

Table 1 shows the mean (across all subjects) of the average DTI-FA, DKI-FA, and DBSI-FA in the WM mask. The mean DKI-FA is significantly lower than both the mean DTI-FA [*t*(11) = −23.32, *p* < 0.001] and the mean DBSI-FA [*t*(11) = −25.26, *p* < 0.001]. The mean DBSI-FA is significantly lower than the DTI-FA [*t*(11) = −5.07, *p* < 0.001]. Figure 1 shows the correlations between DTI-FA, DKI-FA, and DBSI-FA calculated across all subjects and WM voxels. The correlation between DTI-FA and DBSI-FA is the highest (*r*_s_ = 0.90), and the correlation between DKI-FA and DBSI-FA is the lowest (*r*_s_ = 0.78). All correlations are significant at *p* < 0.001.

**Table 1:** **Mean diffusion tensor imaging (DTI)-fractional anisotropy (FA), diffusion kurtosis imaging (DKI)-FA, and diffusion basis spectrum imaging (DBSI)-FA**.

	DTI-FA	DKI-FA	DBSI-FA
Mean	0.43	0.33	0.42
SD	0.01	0.02	0.01

### Relationship among DTI, DBSI, and DKI Parameters

**Figure 2:**
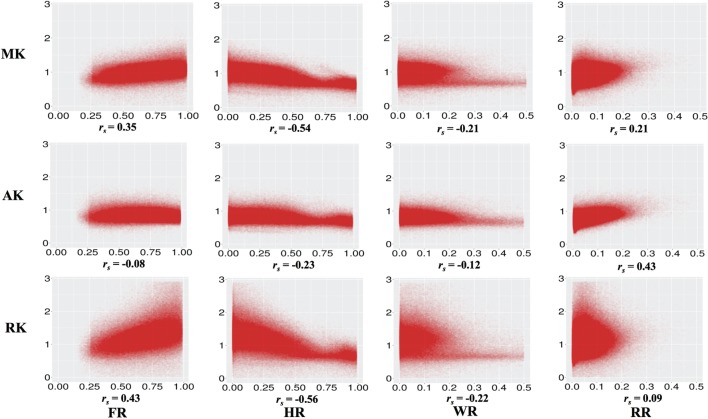
**Correlations between diffusion kurtosis imaging-mean kurtosis (MK)/axial kurtosis (AK)/radial kurtosis (RK) and diffusion basis spectrum imaging-fiber ratio (FR)/hindered ratio (HR)/water ratio (WR)/restricted ratio (RR) across all individuals and white matter voxels**. All *p* values are significant and <0.001.

Figure 2 and Table 2 show the correlations between DKI-MK/AK/RK and DBSI-FR/HR/RR/WR. MK and RK are positively correlated with FR and negatively correlated with HR. AK is positively correlated with the RR.

**Table 2:** **Correlation between diffusion basis spectrum imaging-fiber ratio (FR), hindered ratio (HR), water ratio (WR), and restricted ratio (RR) and diffusion kurtosis imaging-mean kurtosis (MK), axial kurtosis (AK), and radial kurtosis (RK)**.

		FR	HR	WR	RR
MK	*r*_s_	0.35	−0.54	−0.28	0.21
	SD	0.05	0.04	0.06	0.05
AK	*r*_s_	0.08	−0.24	−0.12	0.43
	SD	0.05	0.06	0.05	0.04
RK	*r*_s_	0.44	−0.32	−0.28	0.09
	SD	0.05	0.04	0.05	0.05

**Figure 3:**
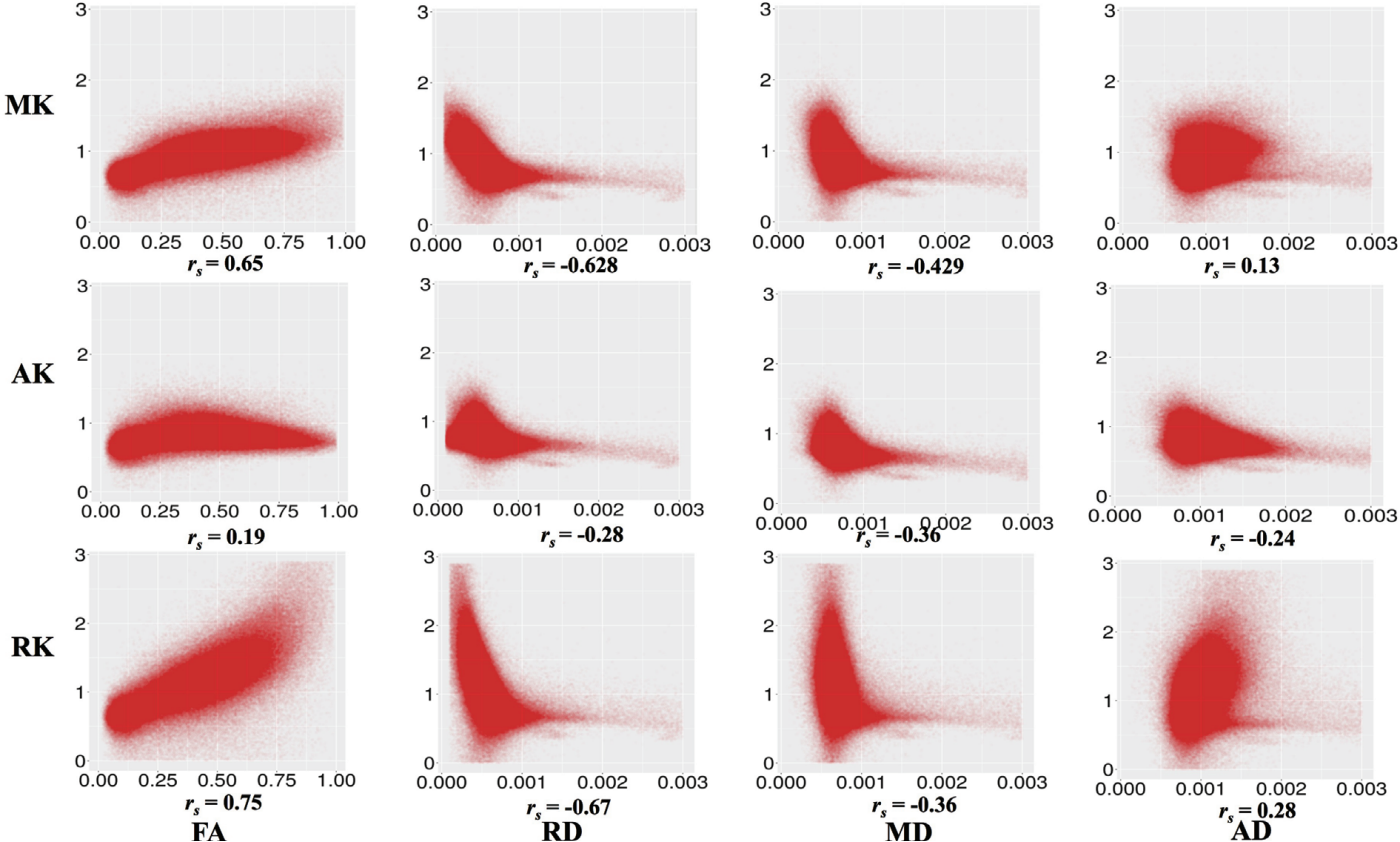
**Correlation between diffusion kurtosis imaging-mean kurtosis (MK)/axial kurtosis (AK)/radial kurtosis (RK) and diffusion tensor imaging-fractional anisotropy (FA)/radial diffusivity (RD)/mean diffusivity (MD)/axial diffusivity (AD) across all individuals and all white matter voxels**. All *p* values are significant and <0.001.

Figure 3 shows the correlation between DKI-MK/AK/RK and DTI-FA/RD/MD/AD. MK and RK are strongly positively correlated with FA and negatively correlated with RD. MD is negatively correlated with MK and RK.

**Figure 4:**
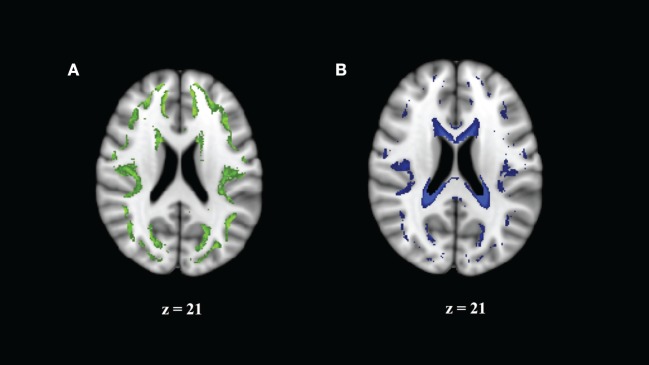
**Regions where correlation of mean kurtosis and diffusion basis spectrum imaging parameters appears to come from a different distribution reflect borderline areas in the white matter (WM) atlas**. **(A)** Hindered ratio >0.75 reflects boundary of GM and WM. **(B)** Water ratio >0.3 reflects partial cerebrospinal fluid volume effects.

We note that higher values of HR or WR seem to represent different relationships to MK, AK, and RK. We examined this by visualizing the average spatial map of voxels with a high HR (>0.75) or a high WR (>0.3) on the MNI 1-mm image (Figure 4). We can see that these values are (Figure 4A) in the border of the white and gray matter, and (Figure 4B) adjacent to CSF, where partial volume effects or atlas misalignment occurs. This indicates that the relationship of DKI and DBSI parameters is different outside of the WM and demonstrates that DBSI can separate different diffusion components that are associated with different types of tissue.

### DBSI Parameters in Regions of High MK

**Figure 5:**
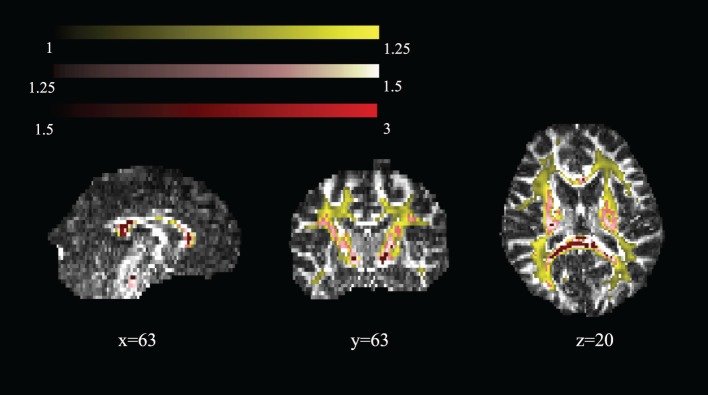
**Spatial map of regions of high mean kurtosis within a representative subject overlaid on the diffusion tensor imaging-fractional anisotropy image (subject 1)**.

Figure 5 shows areas of high MK overlaid on the DTI-FA map for a representative subject. In general, areas with kurtosis greater than 1 are in WM, and areas with kurtosis ranging 1.5–3 are deep in WM (e.g., the CC). Areas of high MK are primarily WM: the mean WM probability where MK > 1 is 0.652 (SD = 0.01). Figure 3 shows that the correlation between FA and MK is *r*_s_ = 0.65, which is verified by noting that the areas of highest MK kurtosis are those with the highest FA.

**Figure 6:**
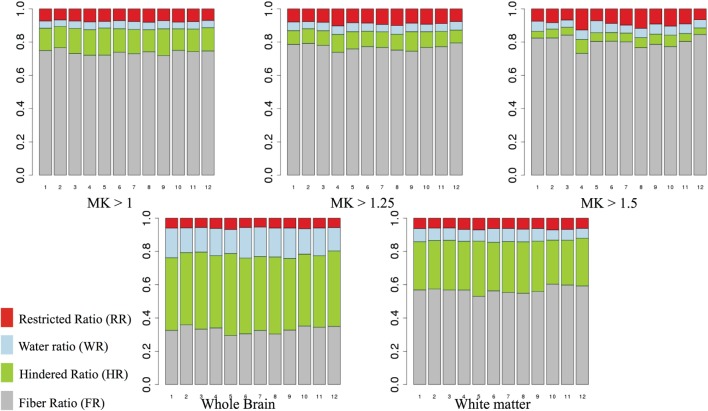
**Mean fiber ratio (FR), hindered ratio (HR), restricted ratio (RR), and water ratio (WR) for areas of high mean kurtosis (MK) (>1, >1.25, and >1.5)**. Gray, fiber ratio; green, hindered ratio; blue, water ratio; red, restricted ratio.

Figure 6 shows the mean FR, HR, RR, and WR in areas of high MK (>1, >1.25, and >1.5) and across the whole brain and WM mask, for each subject (1–12). We note that the relative contribution of the DBSI compartments is quite stable across individuals. As we increase the threshold for MK, we observe that the HR decreases and the FR increases.

**Figure 7:**
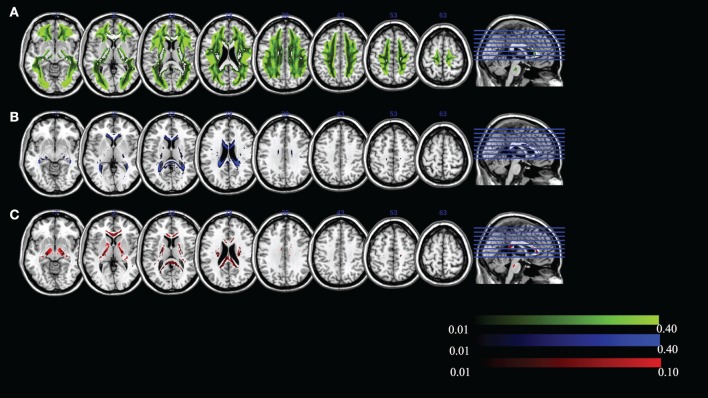
**Maximum ratio images computed in areas of high mean kurtosis excluding fiber ratio for (A) hindered ratio, (B) water ratio, and (C) restricted ratio**. All images are in radiological notation (e.g., left is on the right).

The mean FR dominates in regions of high MK. To examine whether the “residual” values of RR, WR, and HR are meaningful, we visualize the maximum ratio images computed in areas of high MK, excluding FR in Figure 7. We can see that the maximum HR is in areas with high tissue complexity and crossing fibers. The maximum WR is in areas with CSF partial volume effects. Finally, areas with maximum RR occur in deep WM. This finding strongly suggests that multiple local microstructure features contribute to increased MK. Although the relative contribution of RR, WR, and HR is small within areas of high MK, they have a spatial specificity that indicates the underlying tissue composition. Individual subject-specific maps are provided in Figures S4–S15 in Supplementary Material.

**Figure 8:**
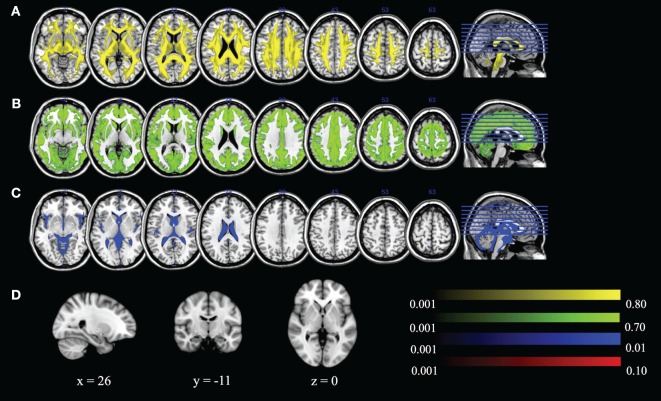
**Maximum ratio images computed across the entire fractional anisotropy standard space mask for (A) fiber ratio, (B) hindered ratio, (C) water ratio, and (D) restricted ratio**. All images are in radiological notation (e.g., left is on the right).

**Figure 9:**
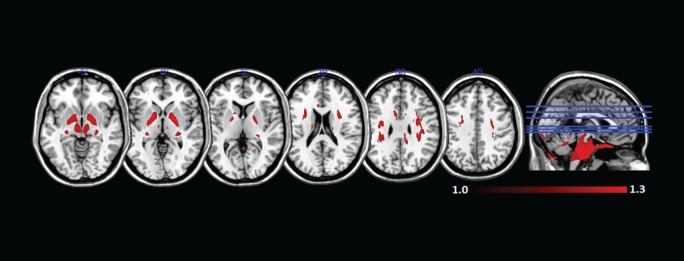
**Mean axial kurtosis (AK) (>1) in standard space across all subjects**. AK is especially high in the globus pallidus and in regions where large white matter fiber tracts intersect.

We can visualize the contribution of each of the DBSI compartments by including FR in the maximum ratio images computed across the entire FA standard space mask (i.e., FR, HR, WR, and RR) as shown in Figure 8. The highest FR is in WM, the highest HR is in gray matter or areas of crossing fibers, and the highest WR is in the ventricles and CSF. There is only a small ROI where mean RR is highest across all subjects, which is in the globus pallidus. Recall that RR and AK are strongly correlated (*r*_s_ = 0.43). The globus pallidus is also a region of unusually high AK; Figure 9 is a spatial map of areas where AK > 1, showing that AK is high in the globus pallidus and in areas where large WM fiber tracts intersect.

### Comparison of Parameters in Areas of Known Fiber Structure

To more closely examine the validity of the DKI and DBSI parameters, we compared DTI, DBSI, and DKI parameters in a region of high crossing fibers and a region of coherent fibers. Table 3 shows the mean and SD of all parameters from all models in these regions.

**Table 3:** **Mean and SD of all parameters from all models in these regions**.

	Coherent fiber (corpus callosum)						Crossing fiber					
	Diffusion tensor imaging (DTI)		Diffusion kurtosis imaging (DKI)		Diffusion basis spectrum imaging (DBSI)		DTI		DKI		DBSI	
	Mean^a^	SD^a^	Mean^a^	SD^a^	Mean	SD	Mean	SD	Mean	SD	Mean	SD
Fractional anisotropy	0.900	0.040	0.803	0.048	0.882	0.034	0.461	0.069	0.411	0.088	0.444	0.086
Mean diffusivity^a^	0.595	0.049	0.876	0.058	0.447	0.035	0.581	0.016	0.700	0.021	0.427	0.010
Axial diffusivity^a^	1.485	0.121	1.952	0.093	1.107	0.096	0.894	0.059	1.028	0.078	0.668	0.056
Radial diffusivity^a^	0.145	0.047	0.338	0.074	0.117	0.028	0.536	0.057	0.536	0.057	0.307	0.030
Mean kurtosis			1.239	0.174					1.208	0.072		
Axial kurtosis			0.763	0.041					1.032	0.086		
Radial kurtosis			2.240	0.559					1.549	0.254		
Fiber ratio					0.076	0.261					0.645	0.093
Hindered ratio					0.020	0.012					0.204	0.070
Water ratio					0.035	0.012					0.037	0.013
Restricted ratio					0.063	0.014					0.113	0.027

*^a^DTI and DKI values are multiplying with 1,000 figure caption*.

We compare parameters produced by each method across the different tissue types. As expected, FA and AD estimates are significantly higher for each method in the coherent fibers than in the crossing fibers, while RD is lower. DKI produces MK, AK, and RK estimates. The MK in the coherent fibers is almost identical to the region of crossing fibers [*t*(11) = 0.76, *p* = 0.461]. The AK is significantly lower in the coherent fibers than the crossing fibers [*t*(11) = −10.49, *p* < 0.001], while the RK is significantly higher [*t*(11) = 5.27, *p* < 0.001]. For DBSI, the DBSI-FR is significantly higher in the coherent fibers than within crossing fibers [*t*(11) = 9.62, *p* < 0.001]. The HR and RR are each significantly lower in the coherent fibers: [*t*(11) = −9.19, *p* < 0.001] and [*t*(11) = −9.19, *p* < 0.001], respectively. There is no significant difference between the WR in the coherent fibers and crossing fibers.

## Discussion

We identified systematic relationships between parameters generated by two different models: DKI and DBSI. Examining DKI–DBSI correlations, we found that MK was positively associated with FR and negatively associated with HR. These relationships were primarily driven by the RK component of MK, especially for HR. This is likely due to the presence of myelin sheaths inducing non-Gaussian diffusion perpendicular to the predominant fiber direction, as well as increasing the proportion of signal from highly restricted and anisotropic compartments (30). AK was robustly and specifically positively correlated with RR. This means that in regions of greater kurtosis along the principal diffusion direction, more signal was assigned to the compartment of isotropic and restricted (low-ADC) diffusion. AK and RR were high in regions with complex fiber crossings, as well as in the globus pallidus. These are both regions of highly interdigitated WM tracts and show complex organization at multiple spatial scales (31). WR was weakly and negatively correlated with the kurtosis metrics. This suggests that fewer microstructural barriers to diffusion results in increased WR and decreased kurtosis, regardless of directionality. In general, non-Gaussian diffusion is reflected through multiple DBSI-assigned compartments. In this healthy cohort, signal from intact axon fibers dominate, and we would expect these relationships to change in the setting of pathology, such as neuroinflammation or edema.

The relationships between DTI and DKI metrics we observed are broadly similar to previous work. This includes a robust positive association between MK and FA (32, 33) and negative associations between MD and MK, RD, and RK, and to a lesser extent AD and AK (34). In this study, DKI-FA is lower than DTI-FA, although this is apparently driven by the smoothing step in preprocessing (see discussion of limitations below).

We found that the spatial distribution of DBSI parameters reflected their biophysical meaning. In the whole brain, FR reflects WM, HR reflects gray matter, and WR reflects CSF. In areas of high MK, the non-FR DBSI components reflect tissue complexity and partial volume effects, where HR is highest in areas of crossing fibers, RR is highest in deep WM, and WR represents partial volume CSF effects.

It is ultimately necessary to validate diffusion models with histological and histopathological data to be sure that the model parameters can be interpreted correctly. In this study, we did not have pathological validation data. Instead, we used ROIs in areas of known anatomical tissue structure (an area of coherent fibers in the genu of the CC and an area of crossing fibers) and compared parameters from the DTI, DKI, and DBSI models in those ROIs. We found that MK did not discriminate region, but the directional kurtosis values AK and RK and the non-WR DBSI measures did. In areas of coherent fibers, AK was lower and RK was higher than in areas of crossing fibers. DBSI parameters also systematically discriminated tissue structure. DBSI-FR is higher, and HR and RR were lower in areas of coherent fibers than in areas of crossing fibers.

This study has limitations. The acquisition in this study was developed specifically for DKI analysis. DBSI is relatively flexible in terms of acquisition protocol with multiple diffusion weightings. A composite diffusion protocol, developed for both DKI and DBSI, would be better to further confirm our preliminary findings in this study. Nevertheless, a strength of this paper is that we were able to compare different models on the same acquisition. We used pipelines optimized for each type of processing (DTI, DBSI, and DKI, respectively). Gaussian smoothing was applied to the diffusion data only for the DKI pipeline, as is typical (21–23). We also repeated our DKI analyses without smoothing, and it did not change the relationship of the DKI parameters to DBSI or DTI parameters (see Supplementary Material). For higher-order methods, such as DKI and DBSI, the signal quality (signal to noise ratio) is critical for accurate computation and interpretation. High *b*-values (>2,000) that are noisy are typically thought to distort DKI quadratic fitting, and it is common practice to exclude them (35, 36) However, exclusion of *b*-values >2,000 did not change the relationship of DKI parameters to DBSI or DTI parameters (see Supplementary Material). Although we examined these important preprocessing parameters, an exhaustive study of the effects of preprocessing or noise was beyond the scope of our study. We did not collect a T1-weighted image for these subjects. This limited our ability to restrict analysis to areas of WM measured independently from T1 segmentation, as opposed to being identified from a standard space atlas. We observed that some voxels within our atlas-derived WM mask fell within gray matter or ventricles and had a different relationship to kurtosis parameters. This may have altered the values of our correlations. Finally, we could not directly compare the discriminatory ability of the DKI and DBSI parameters in identifying tissue complexity without a cross-validation sample.

Overall, these results enable a sounder comparison of the DKI and DBSI literature. For example, there has been recent work showing that HR is increased in MS lesions, compared to control regions (17), and that MK is decreased in patients with MS, compared to controls (37). These two results may be considered concordant with respect to the empirical relationship between HR and MK seen here. Ultimately, a model is just a way to characterize the underlying biological system. Although DBSI showed meaningful biological specificity, DKI may also provide similar information but expressed in less interpretable parameters. Pathological validation of model parameters and evaluation of their discriminatory capabilities are necessary to advance the use of higher dimensional diffusion models.

## Author Contributions

Study conception and design: SW, YW, QW, WL, TG, and TM. Analysis and interpretation of data: SW, DP, YW, QW, TM, and TG. Drafting of manuscript: all. Critical revision: all.

## Conflict of Interest Statement

The authors declare that the research was conducted in the absence of any commercial or financial relationships that could be construed as a potential conflict of interest.

## Footnotes

^1^http://github.com/danjonpeterson/dti_preproc.

^2^http://nipy.org/dipy.
