# Supplementary material for: Empirical Comparison of Diffusion Kurtosis Imaging and Diffusion Basis Spectrum Imaging Using the Same Acquisition in Healthy Young Adults
Source: Front Neurol. 2017 Mar 29;8:118. doi: 10.3389/fneur.2017.00118 (PMC5372828; doi:10.3389/fneur.2017.00118)
Supplement: Supplementary file 1 [file data_sheet_1.pdf]

Supplemental Material

1. Location of ROIs with coherent fibers and crossing fibers

Supplemental Figure 1 shows two regions of interest (ROIs) for one individual (Subject 1) overlaid on the DTI-FA map. A) The first ROI is deep in the genu of the corpus callosum (CC), a coherent fiber bundle. B) The second ROI is in the decussation of the corpus callosum and the inferior fronto-occipital fasciculus (IFOF), an area of crossing fibers.

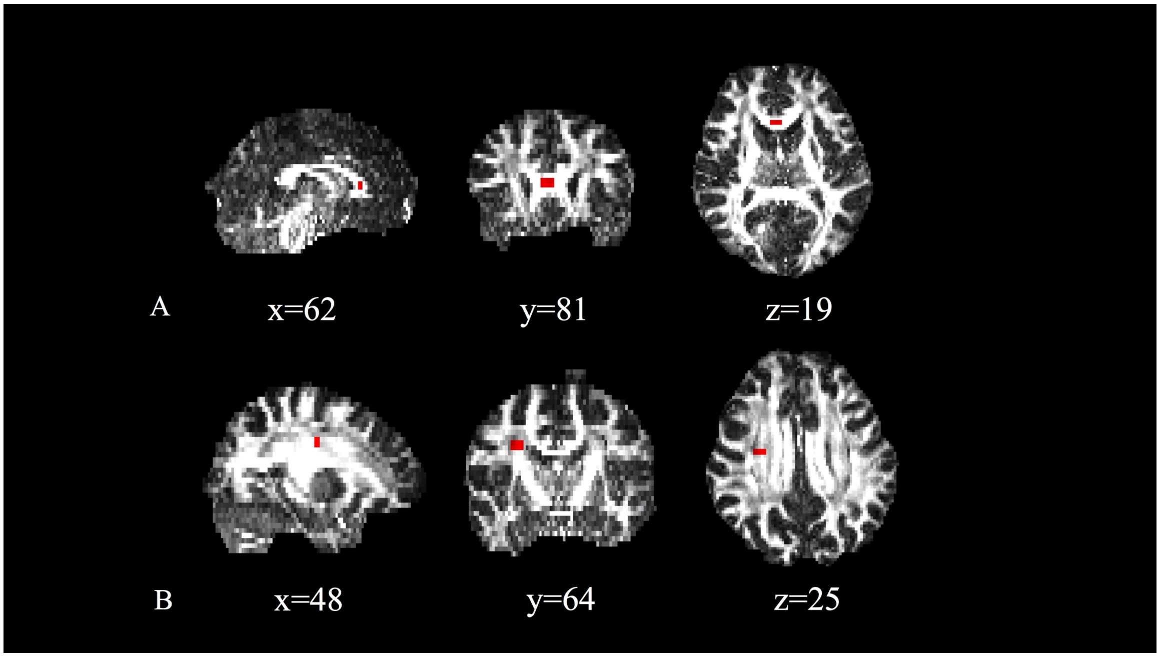

Supplemental Figure 1. Regions of interest shown on the DTI-FA map of subject 1. (A) Coherent fiber ROI (B) Crossing fiber ROI.

2. Maximum ratio maps of DBSI-HR, RR, WR overlaid on DTI-FA maps

Supplemental Figures 2-13 show the maximum of DBSI-HR (green), DBSI-RR (red), and DBSI-WR(blue) in each voxel, excluding DBSI-FR, and overlaid with DTI FA maps for each individual.

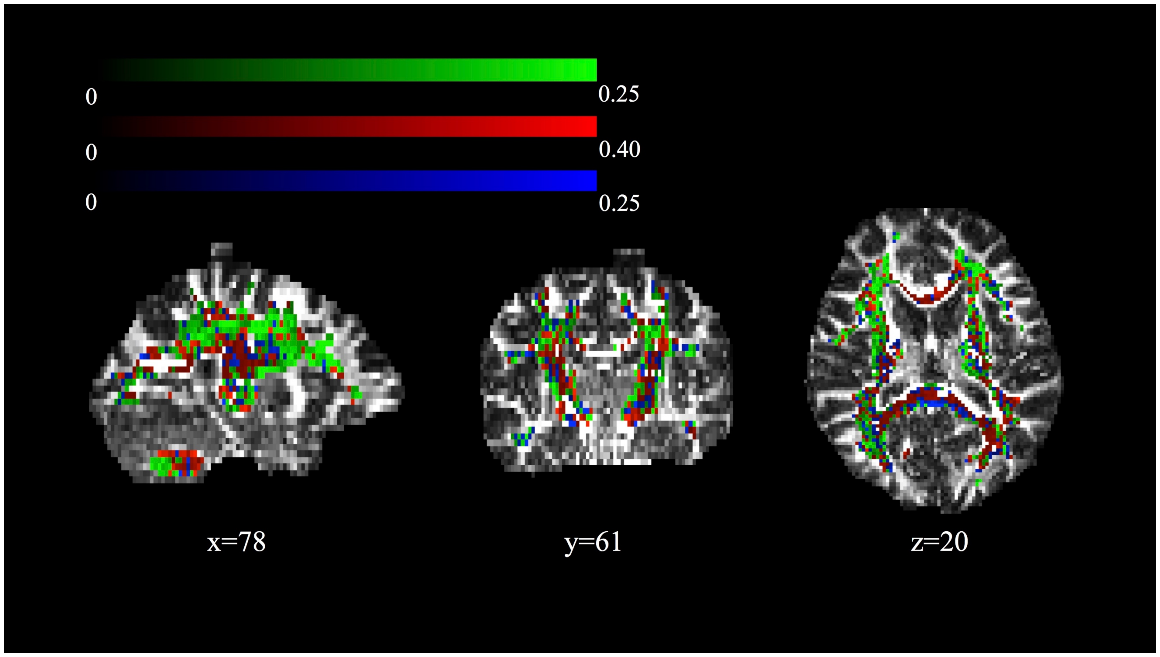

Supplemental Figure 2. Maximum ratio map for Subject 1. Green=HR, Red=RR, Blue=WR

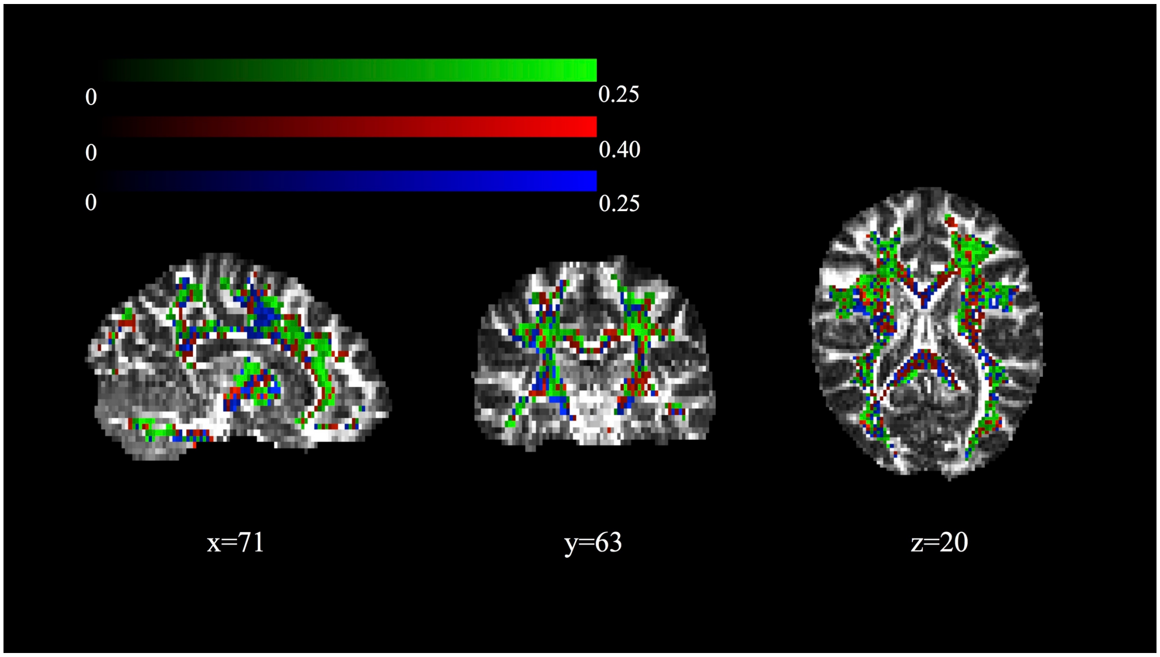

Supplemental Figure 3. Maximum ratio map for Subject 2. Green=HR, Red=RR, Blue=WR

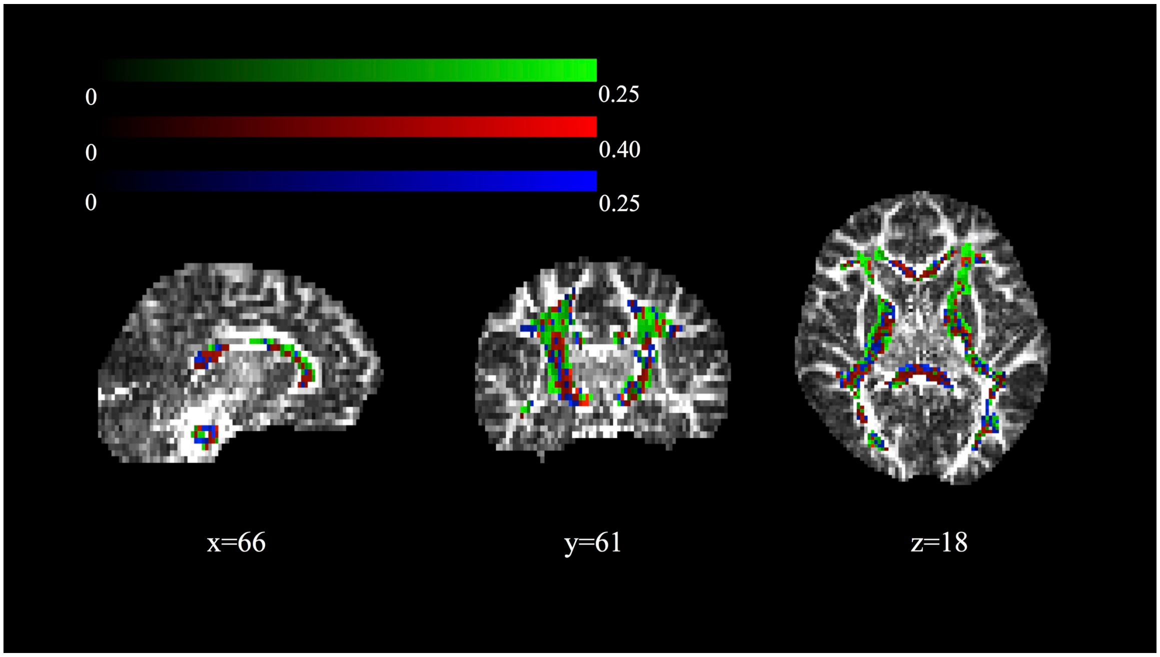

Supplemental Figure 4. Maximum ratio map for Subject 3. Green=HR, Red=RR, Blue=WR

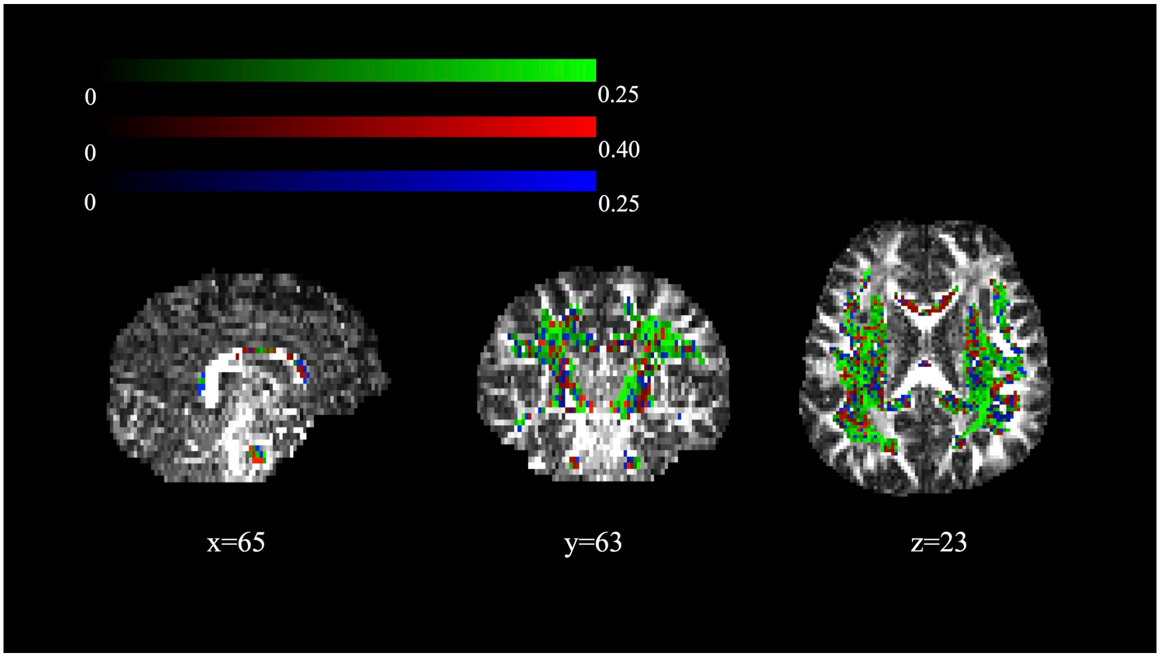

Supplemental Figure 5. Maximum ratio map for Subject 4. Green=HR, Red=RR, Blue=WR

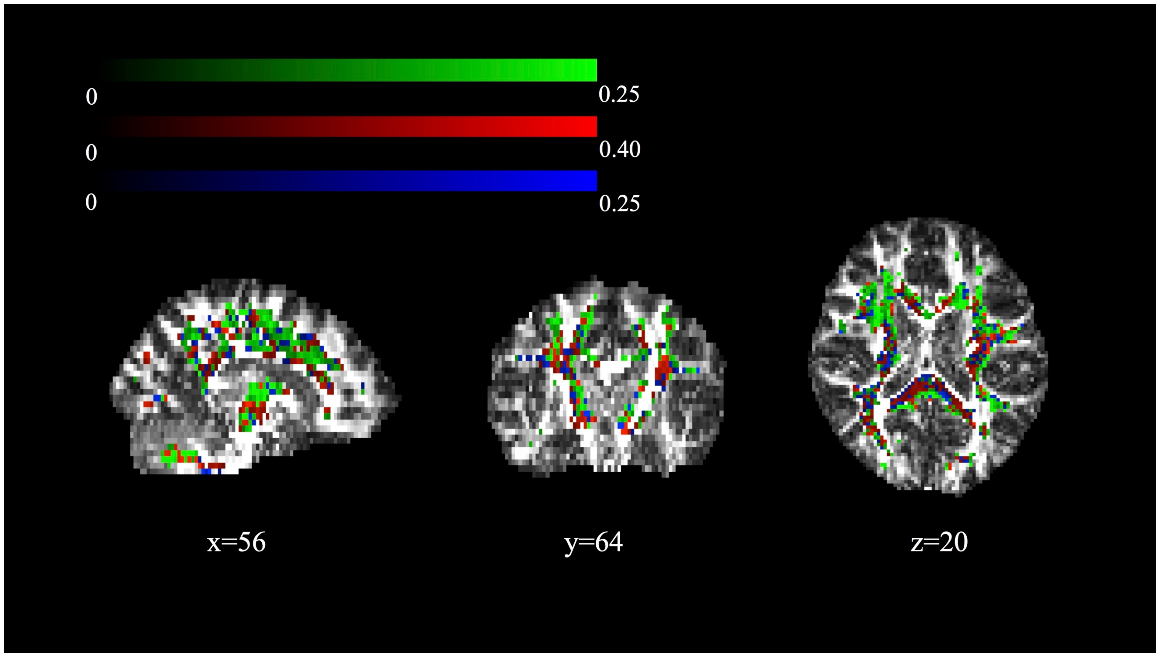

Supplemental Figure 6. Maximum ratio map for Subject 5. Green=HR, Red=RR, Blue=WR

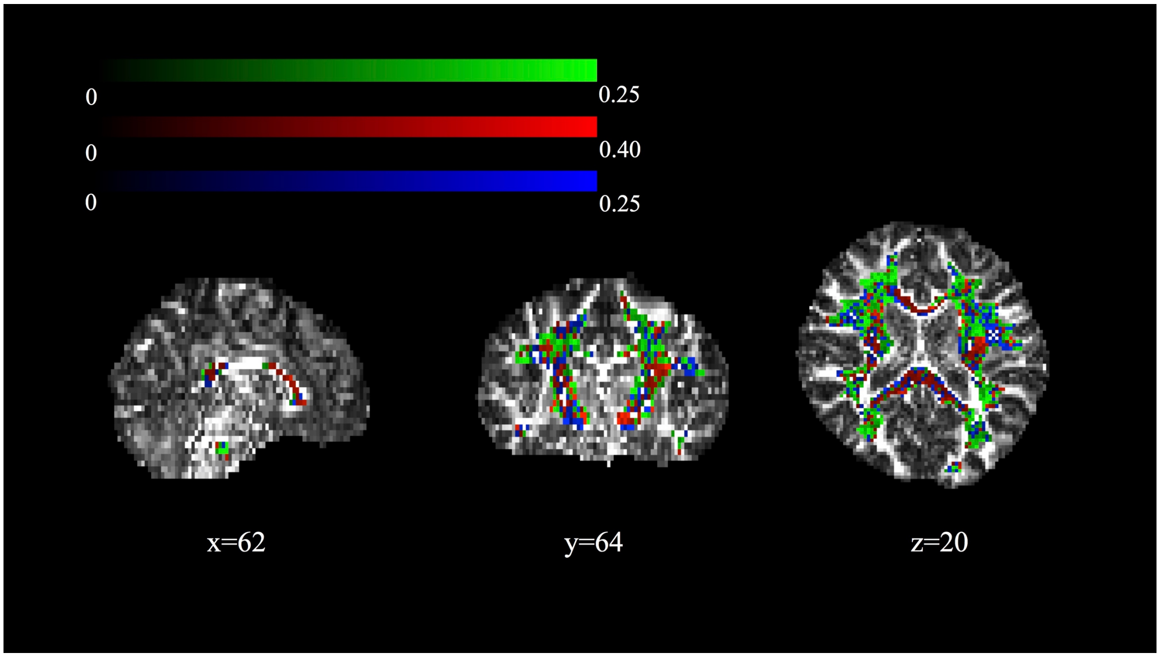

Supplemental Figure 7. Maximum ratio map for Subject 6. Green=HR, Red=RR, Blue=WR

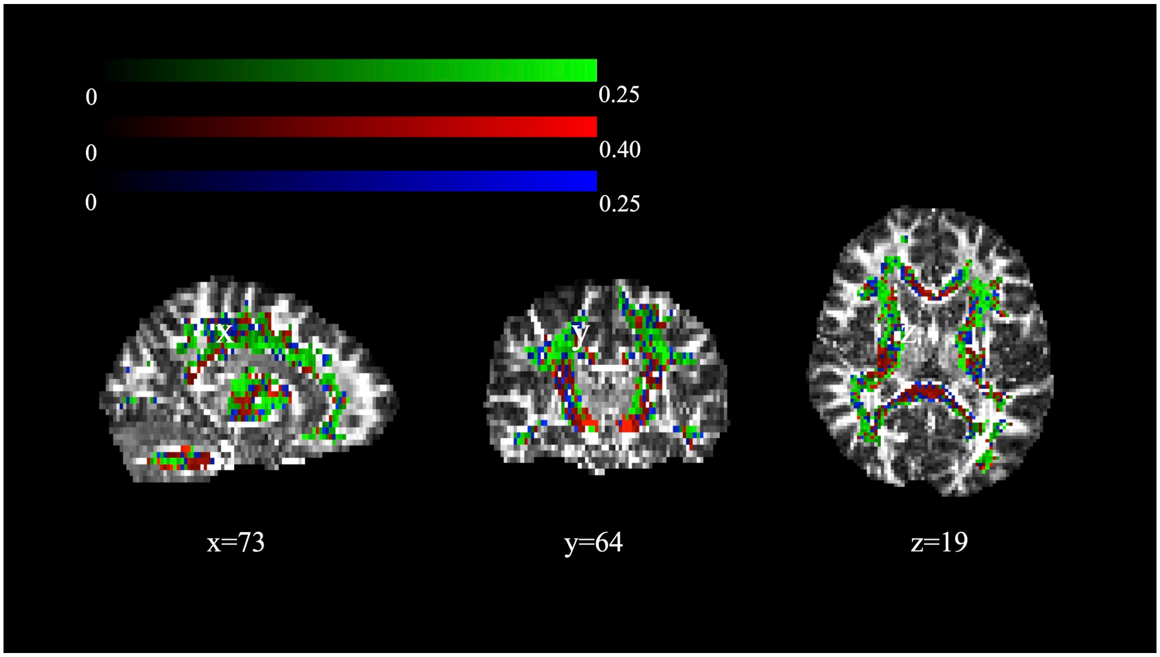

Supplemental Figure 8. Maximum ratio map for Subject 7. Green=HR, Red=RR, Blue=WR

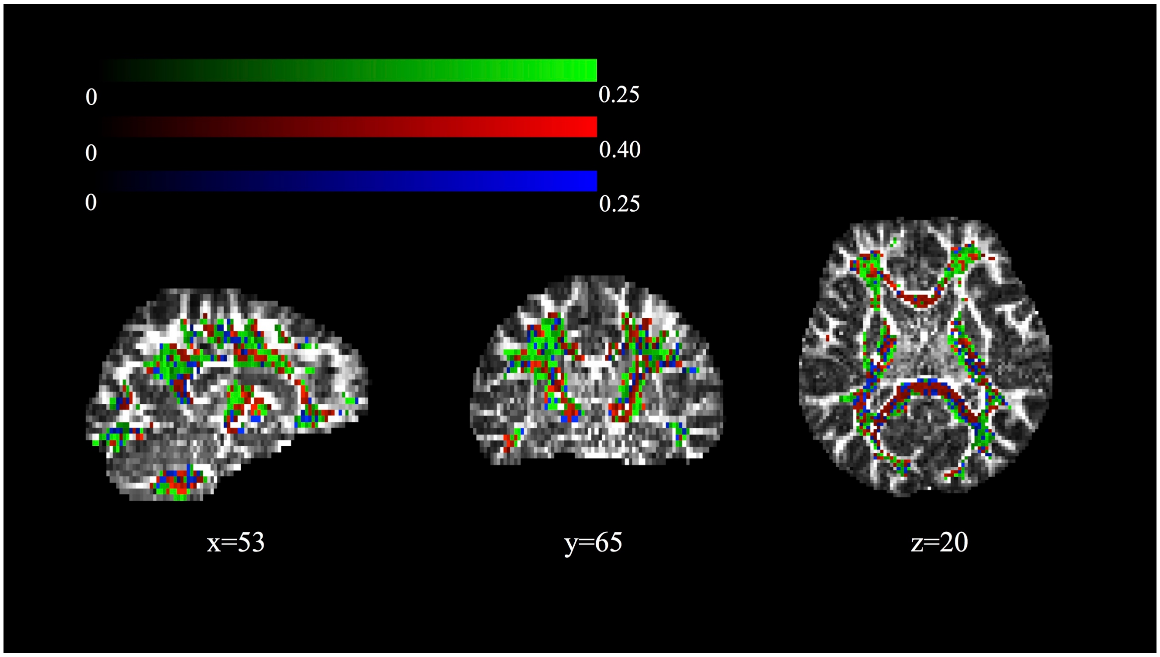

Supplemental Figure 9. Maximum ratio map for Subject 8. Green=HR, Red=RR, Blue=WR

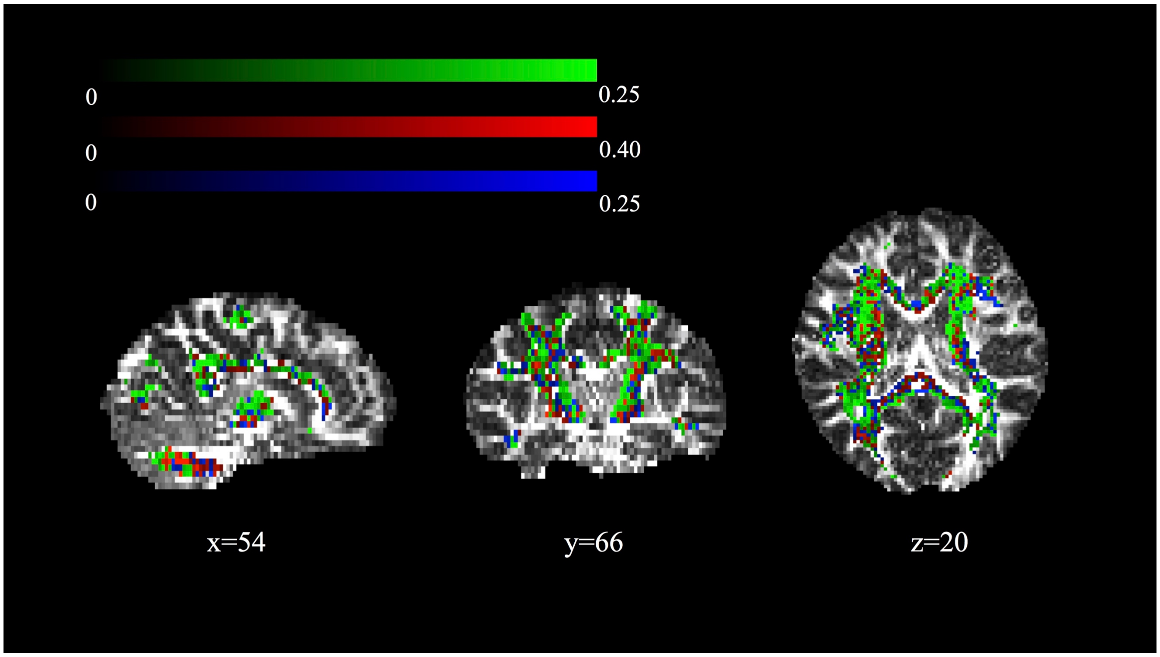

Supplemental Figure 10. Maximum ratio map for Subject 9. Green=HR, Red=RR, Blue=WR

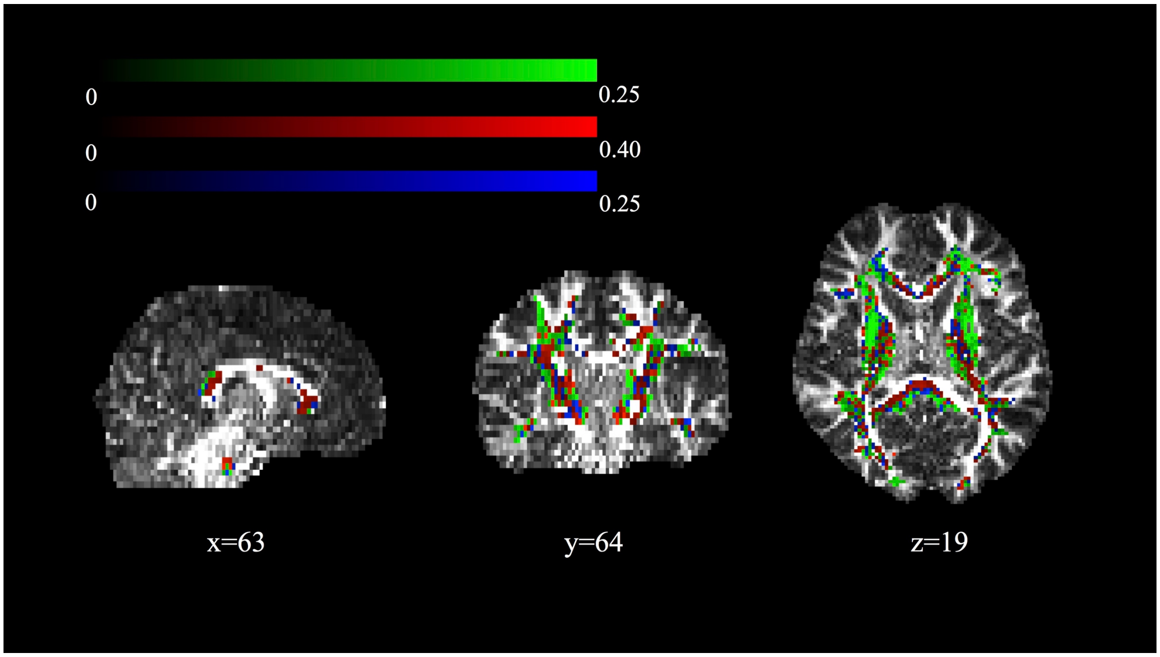

Supplemental Figure 11. Maximum ratio map for Subject 10. Green=HR, Red=RR, Blue=WR

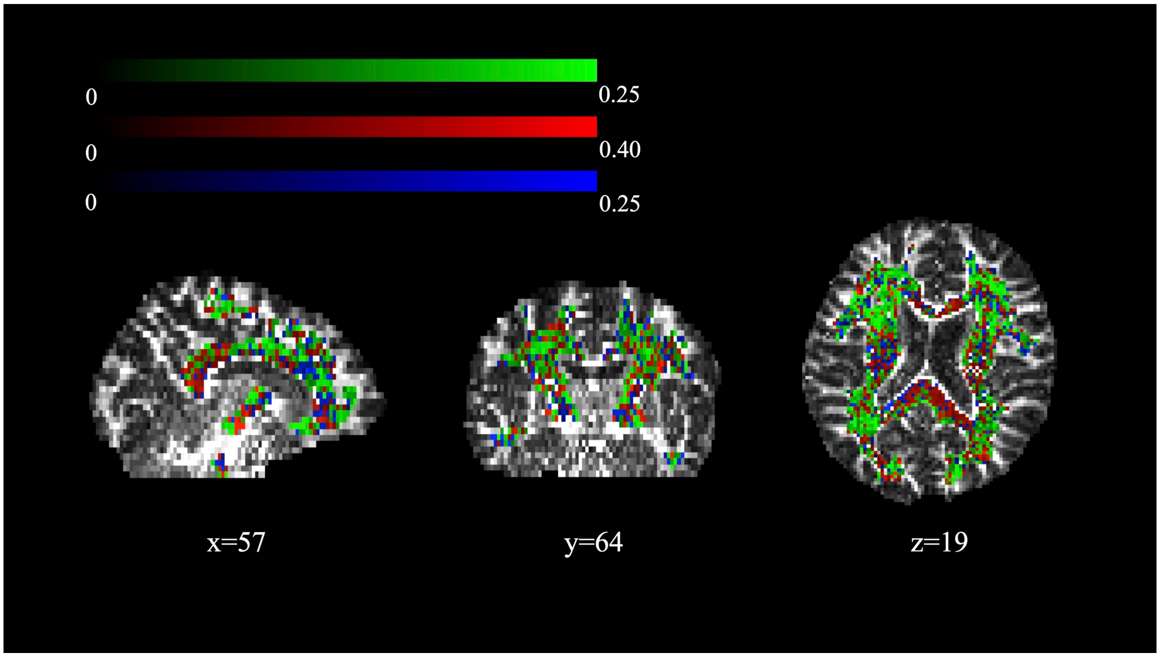

Supplemental Figure 12. Maximum ratio map for Subject 11. Green=HR, Red=RR, Blue=WR

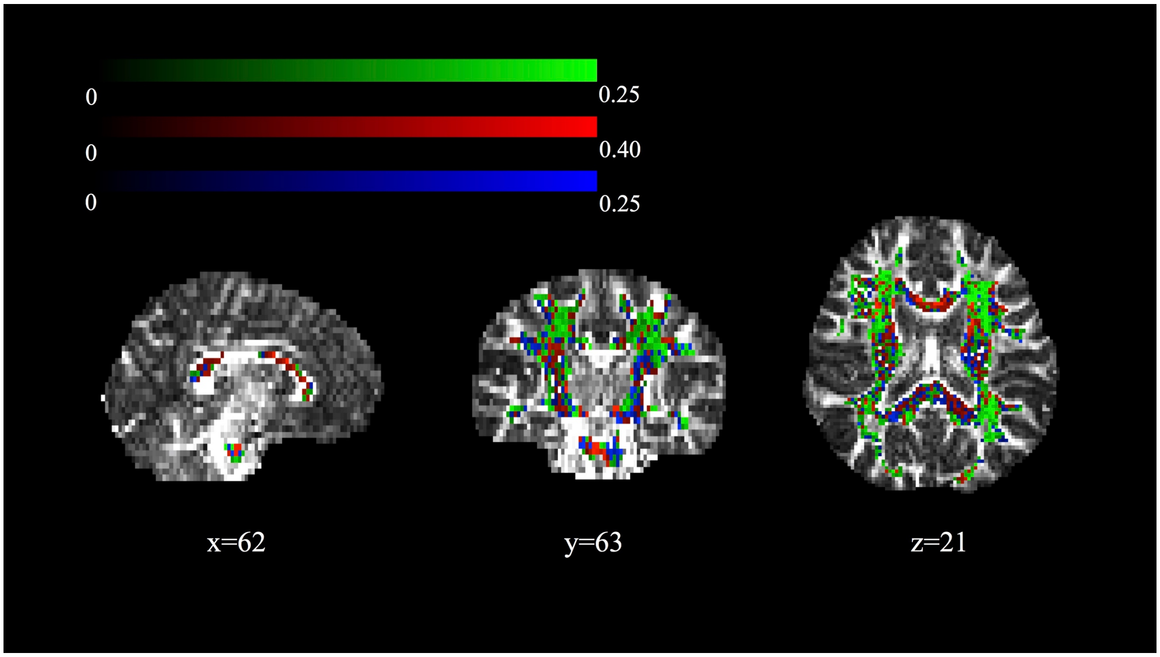

Supplemental Figure 13. Maximum ratio map for Subject 12. Green=HR, Red=RR, Blue=WR

3. Relationship between DTI, DBSI and unsmoothed DKI parameters, and DKI computed with b values no greater than 2000

Supplemental Figure 14 shows the correlations between DTI-FA, unsmoothed DKI-FA and DBSI-FA calculated across all subjects and white matter voxels. Supplemental Figure 15 shows the correlation between unsmoothed DKI-MK, AK, RK and DBSI-FR, HR, WR, RR. Supplemental Figure 16 shows the correlations between DTI-FA, DKI-FA with b values no greater than 2000, and DBSI-FA calculated across all subjects and white matter voxels. Supplemental Figure 17 shows the correlations between DTI-FA, DKI-FA computed using b value no greater than 2000 and DBSI-FA calculated across all subjects and white matter voxels.

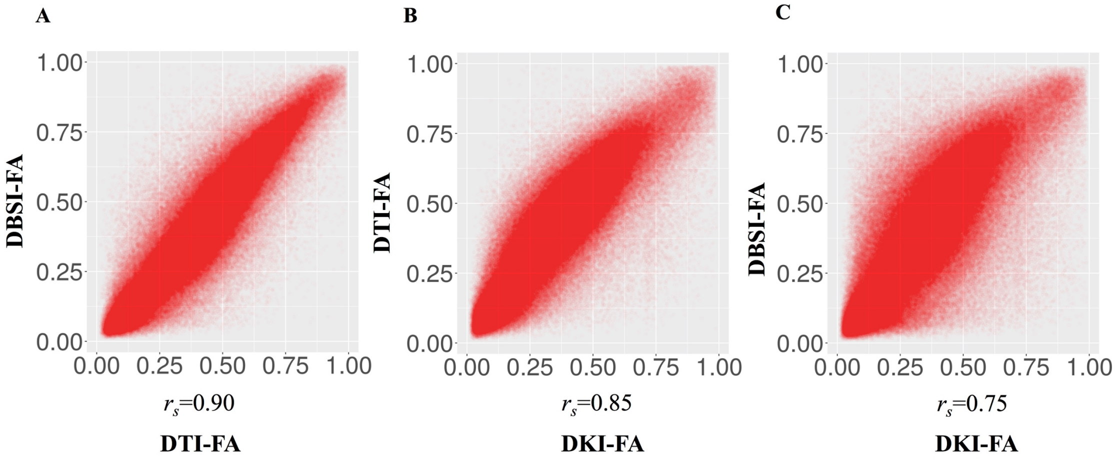

Supplemental Figure 14. Correlation between FA calculated from different models across all individuals and WM voxels using unsmoothed DKI data. All p values are significant and < 0.001.

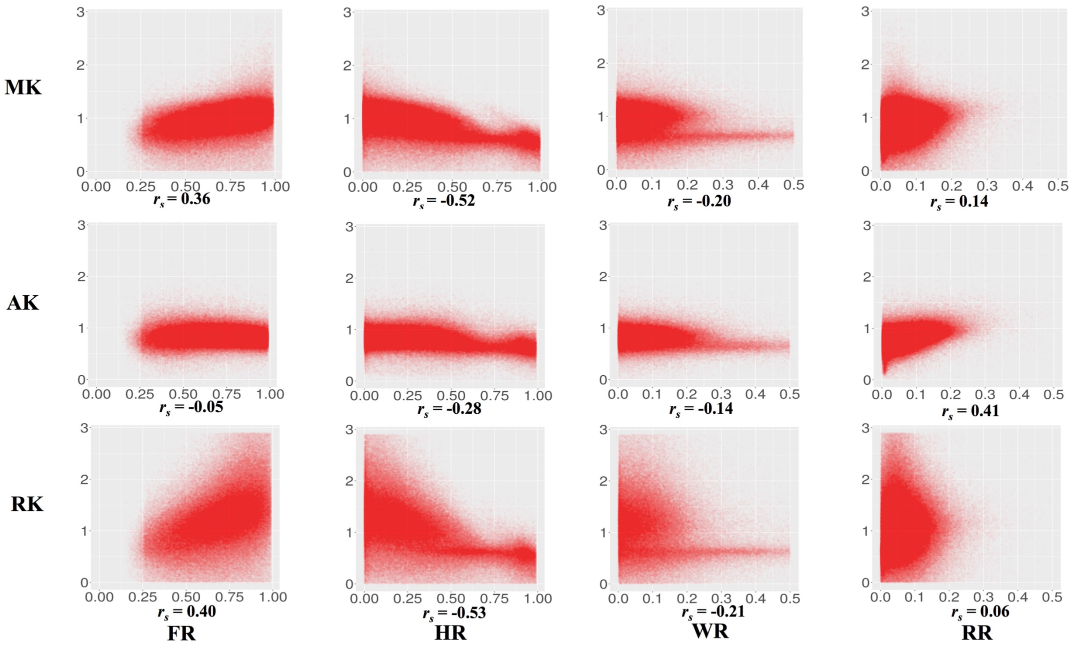

Supplemental Figure 15. Correlations between unsmoothed DKI-MK/AK/RK and DBSI-FR/HR/WR/RR across all individuals and WM voxels. All p values are significant and < 0.001.

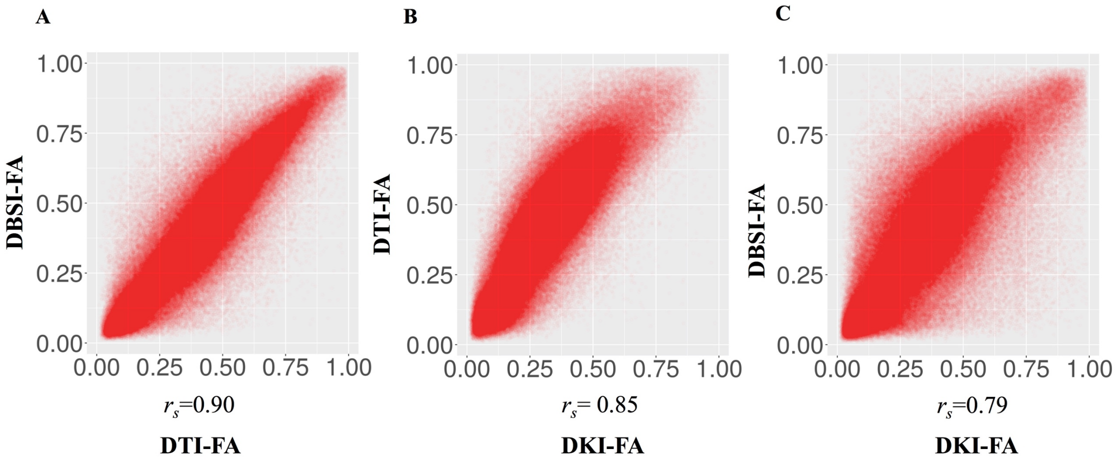

Supplemental Figure 16. Correlation between FA calculated from different models across all individuals and WM voxels using DKI data with b value no greater than 2000. All p

values are significant and < 0.001.

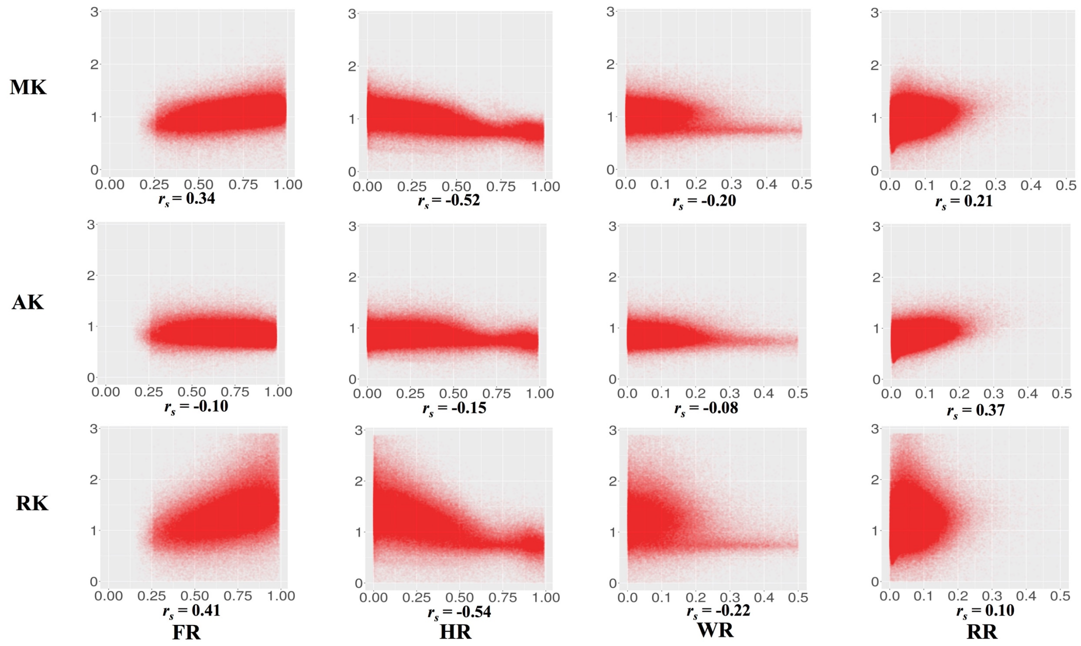

Supplemental Figure 17. Correlation between DKI-MK/AK/RK/ with b value no greater than 2000 and DTI-FA/RD/MD/AD across all individuals and all WM voxels. All p values are significant and < 0.001.
