# Supplementary material for: Empirical Comparison of Diffusion Kurtosis Imaging and Diffusion Basis Spectrum Imaging Using the Same Acquisition in Healthy Young Adults
Source: Front Neurol. 2017 Mar 29;8:118. doi: 10.3389/fneur.2017.00118 (PMC5372828; doi:10.3389/fneur.2017.00118)
Supplement: Supplementary file 2 [file data_sheet_2.pdf]

Makefile Documentation

Contents

1 This File 2 Targets 3 Variables 4 Intermediate Files 5 Makefiles

1 This File

- This document was prepared on January 6, 2017 at 18:56 by `sjwang21`.
- Prepared in: `/mnt/home/djpeters/Public/Trevor/makefiles_for_dbsi_dki_comp`
- This file documents the following makefiles:
  `dti_preproc.mk kurtosis.mk kurtosis_unsmoothed.mk kurtosis_nob2500.mk probability_high_kurtosis.mk registration.mk reorientation_dbsi.mk dbsi_-mask.mk correlogram.mk`

1.1 Description

This is a document describing the makefiles and targets for a project comparing DBSI and DKI models of diffusion by fitting them to the same data.

From the manuscript:

As Diffusion Tensor Imaging (DTI) gains widespread use, many researchers have been motivated to go beyond the tensor model and fit more complex diffusion models, in the hope of gaining a more complete description of white matter microstructure. Two such models are Diffusion Kurtosis Imaging (DKI) and Diffusion Basis Spectrum Imaging (DBSI). It is not clear which DKI parameters are most closely related to DBSI parameters, so in the interest of enabling comparisons between DKI and DBSI studies, we have conducted an empirical survey of the inter-relation of these models in 12 healthy volunteers. We found that Mean Kurtosis (MK) is positively associated with the DBSI Fiber Ratio, and negatively associated with the Hindered Ratio. This was primarily driven by the radial component of MK. Axial Kurtosis was strongly and specifically correlated with the Restricted Ratio. The joint spatial distributions of DBSI and DKI parameters are tissue-depenent and stable across healthy individuals.

1.2 Files

`dti_preproc.mk` DTI preprocessing including motion correction using eddy, tensor fitting using FSL in fit_tensor.sh to calculate FA, AD, RD maps (https://github.com/danjonpeterson/dti_-preproc)

`kurtosis.mk` DKI preprocessing - takes motion-correctied diffusion data and solves the DKI model, using the python package 'dipy'

kurtosis_unsmoothed.mk DKI preprocessing - takes unsmoothed motion-correctied diffusion data and solves the dki model, using the python package 'dipy'

kurtosis_nob2500.mk DKI preprocessing - takes motion-correctied diffusion data with b value no greater than 2500 and solves the dki model, using the python package 'dipy'

probability_high_kurtosis.mk Compute the probablity of white matter in regions of high MK

registration.mk Using ANTs(Advanced Normalization Tools) to register DTI-FA, DBSI-FA and MK to standard space, transform white matter mask and brain mask in standard space into subject specific space

reorientation_dbsi.mk Reorder the data storage to permit changes between axial,sagital and coronal slicing to change the orientation information in the header as well as reorerding the data: delete the orientation information and reset the orientation information by copying the geometric information form DTI FA maps

dbsi_mask.mk Use FSL to mask out DBSI water(WR), hindered(HR) and restricted(RR) ratio maps with high MK mask and compute the maximum of water, hindered and restricted ratio maps for each individual; Use ANTs to register fiber, water, hindered and restricted maps and WR,HR and RR masked out by high MK mask to standard FA map in 1mm space

correlogram.mk Use FSL to mask out DTI, DKI, DBSI parameters that will be used to compute the correlation between the three models with white matter mask and brain mask in subject specific space

Note that the items are sorted uppercase, then lowercase: [A-Za-z]

2 Targets

Target	Description
kurtosis (kurtosis.mk)	Do all kurtosis processing using DKI model with fit_kurtosis.py to generate MK/AK/RK maps and using FSL to threshold MK/AK/RK maps from 0-3
kurtosis (kurtosis_nob2500.mk)	Do all kurtosis processing with b0 values no greater than 2500 using DKI model with fit_kurtosis.py to generate MK/AK/RK maps and using FSL to threshold MK/AK/RK maps from 0-3
kurtosis-unsmoothed (kurtosis_unsmoothed.mk)	Do all kurtosis processing using DKI model with fit_kurtosis.py to generate unsmoothed MK/AK/RK maps and using FSL to threshold unsmoothed MK/AK/RK maps from 0-3

kurtosis_dir (kurtosis.mk)	Create the the output directory for kurtosis fitting
kurtosis_dir (kurtosis_nob2500.mk)	Create the output directory for kurtosis fitting removing b=2500 images
kurtosis_probability (probability_high_-kurtosis.mk)	Make a high MK mask(mask out with whiter matter mask in subject specific space and brain mask in subject specific space with threshold of MK>1) and use ANTs to register high MK mask to standard space, mask out white matter prior image in standard space with the high MK mask in standard space
kurtosis_us_dir (kurtosis_unsmoothed.mk)	Create the unsmoothed kurtosis directory
probab_dir (probability_high_-kurtosis.mk)	Make a directory that will contain high MK mask, high MK mask in standard space, white prior in standard space masking out by high MK mask in standrad space
reg-dir (registration.mk)	Make a directory for images registered to standard space
register-all (registration.mk)	Erode the FA map a little for good registration and register dti_FA/MK/DBSI-FA to standard space
tensor (dti_preproc.mk)	Fit the tensor using DTI model using fit_tensor.sh to calculate dti_FA, dti_AD and dti_RD
transform (registration.mk)	Using ANTs to transform white matter mask and brain mask from standard space into subject specific space to mask out images in subject specific space

3 Variables

Note: Variables with an asterisk ("*") are global variables initialized with "export." Read more about exporting variables in the GNU Make manual: 5.7.2 Communicating Variables to a Sub-make

Variable	Definition & Description
* OMP_NUM_THREADS (dti_preproc.mk)	1<br>Set open MP number of threads to be 1, so that we can parallelize using make. (available to sub-makes)
ANTSpath (registration.mk)	/usr/local/ANTs-2.1.0-rc3/bin<br>The location of ANTs(Advanced Normalization Tools).

BINDIR (dti_preproc.mk)	$(PROJECT_HOME)/bin<br>Directory that contains the executable programs used in this workflow
DTIOUTDIR (dti_preproc.mk)	dti<br>Name of the output directory for FA,MD,AD,RD maps processed using standard DTI model
OUTDIR (kurtosis.mk)	kurtosis<br>Name the the output directory used for DKI processing
PROJECT_HOME (dti_preproc.mk)	/mnt/adrc/dki-dbsi<br>Top dirctory that contains all files and directory in this workflow
SIGMA (kurtosis.mk)	1.6986<br>4mm FWHM (fwhm = 2.3548 * sigma (smoothing factor for kurtosis data))
STDBRAIN (registration.mk)	/usr/share/fsl/data/standard/FMRIB58_FA_-1mm.nii.gz<br>Standard FA image in 1mm space
WMATLAS (probability_high_-kurtosis.mk)	/mnt/adrc/dki-dbsi/atlas/avg152T1_white.nii.gz<br>Standard T1 white matter image

4 Intermediate Files

$DTIOUTDIR/dti_AD.nii.gz dti_preproc.mk

Calculate AD image from eigenvalues from DTI processing. Scales image to be consistent with MD

$DTIOUTDIR/dti_FA.nii.gz dti_preproc.mk

Fit the tensor using DTI model with fit_tensor.sh to generate FA maps

$DTIOUTDIR/dti_RD.nii.gz dti_preproc.mk

Calculate RD image from eigenvalues from DTI processing. Scales image to be consistent with MD

$DTIOUTDIR/mc_raw_diffusion.nii.gz dti_preproc.mk

Do motion correction using eddy in motion_correct.sh for DTI model

atlas_brain_mask_tosubj.nii.gz registration.mk

Brain image in subject specific space using ANTs

atlas_wm_mask_tosubj.nii.gz registration.mk
White matter mask in subject specific space using ANTs

dbsi.reorient/raw_dti_fa_map_ero.nii.gz registration.mk
Eroded DBSI FA map a little with FSL for good registration

dbsi.reorient/raw_dti_fa_map_ss.nii.gz registration.mk
Masked out DBSI FA map with brain mask

dti/dti_FA_ero.nii.gz registration.mk
Eroded DTI FA map a little with FSL for good registration

kurtosis/MK.nii.gz kurtosis_nob2500.mk
MK map with b0 values no greater than 2000

kurtosis/MK.nii.gz kurtosis.mk
MK map from DKI processing

kurtosis/MK_thresh.nii.gz kurtosis_nob2500.mk
Thresholded MK map(0-3) with b0 value no greater than 2000

kurtosis/MK_thresh.nii.gz kurtosis.mk
Thresholded MK(0-3)

kurtosis/sm_mc_raw_diffusion.nii.gz kurtosis_nob2500.mk
Smoothed motion-corrected diffusion data used in DKI pipeline with b0 values no greater than 2000

kurtosis/sm_mc_raw_diffusion.nii.gz kurtosis.mk
Smooth motion-corrected diffusion data with smoothing factor

kurtosis/sm_mc_raw_diffusion_nob2500.nii.gz kurtosis_nob2500.mk
Remove b=2500 images from smoothing diffusion data

kurtosis_probability/avg_wm_high_MK_masked.nii.gz probability_high_kurtosis.mk
White prior image in standard space masked out with high MK mask in standard space using FSL

kurtosis_probability/high_MK_mask.nii.gz probability_high_kurtosis.mk
High MK mask using FSL to mask out MK with whiter matter mask in subject specific space and brain mask in subject specific space with threshold of MK>1

kurtosis_probability/high_MK_mask_tostd.nii.gz probability_high_kurtosis.mk
High MK mask registered to standard FA 1mm image using ants and upscaled into 2mm with flirt to be consistent with white prior image in standard space

kurtosis_unsmoothed/MK.nii.gz kurtosis_unsmoothed.mk
MK file created from DKI pipeline on unsmoothed data

kurtosis_unsmoothed/MK_thresh.nii.gz kurtosis_unsmoothed.mk
Thresholded (0-3) measures and generate MK map generated from DKI pipeline in unsmoothed data

kurtosis_unsmoothed/MK_thresh_masked.nii.gz kurtosis_unsmoothed.mk
MK map(0-3) masked out with white matter mask and brain mask in subject specific space

standard-space/AK_tostd.nii.gz registration.mk
DKI AK map in stardard space using ANTs

standard-space/FAtostandardWarped.nii.gz registration.mk
DTI FA map in standard space using ANTs

standard-space/MK_tostd.nii.gz registration.mk
DKI MK map in standard space using ANTs

standard-space/dbsi_fa_tostd.nii.gz registration.mk
Eroded DBSI FA map in standard space using ANTs

5 Makefiles

5.1 dti_preproc.mk

```
#* DTI preprocessing including motion correction using eddy, tensor fitting using
   FSL in fit_tensor.sh to calculate FA, AD, RD maps  (https://github.com/
   danjonpeterson/dti_preproc)
# This makefile runs through the DTI preprocessing pipleline

# file name conventions:

# INPUT FILES
# raw_diffusion.nii.gz
# native_brain_mask.nii.gz
# raw_diffusion_bval.txt
# raw_diffusion_bvecs.txt

#! Top dirctory that contains all files and directory in this workflow
PROJECT_HOME=/mnt/adrc/dki-dbsi

#! Directory that contains the executable programs used in this workflow
BINDIR=$(PROJECT_HOME)/bin

#! Name of the output directory for FA,MD,AD,RD maps processed using standard DTI
   model
DTIOUTDIR=dti

#! Set open MP number of threads to be 1, so that we can parallelize using make.
export OMP_NUM_THREADS=1

.PHONY: tensor

#? Keep everything by default
.SECONDARY:

define dti_usage
	@echo
	@echo
	@echo Usage:
	@echo "make fit_tensor          Makes the fa and tensor images"
	@echo "make clean               Removes everything except for the source
	   data"
	@echo "make mostlyclean         Removes intermediate files"
	@echo
	@echo
endef

#? Fit the tensor using DTI model using fit_tensor.sh to calculate dti_FA, dti_AD
   and dti_RD
tensor: ${DTIOUTDIR}/dti_FA.nii.gz ${DTIOUTDIR}/dti_RD.nii.gz ${DTIOUTDIR}/dti_AD.
   nii.gz

#> Do motion correction using eddy in motion_correct.sh for DTI model
${DTIOUTDIR}/mc_raw_diffusion.nii.gz: raw_diffusion.nii.gz raw_diffusion_bval.txt
   raw_diffusion_bvec.txt native_brain_mask.nii.gz
```

```
        ${BINDIR}/dti_preproc/motion_correct.sh -k raw_diffusion.nii.gz  -b
           raw_diffusion_bval.txt -r raw_diffusion_bvec.txt -M native_brain_mask.
           nii.gz -o ${DTIOUTDIR}

#> Fit the tensor using DTI model with fit_tensor.sh to generate FA maps
${DTIOUTDIR}/dti_FA.nii.gz: ${DTIOUTDIR}/mc_raw_diffusion.nii.gz
   raw_diffusion_bval.txt
        ${BINDIR}/dti_preproc/fit_tensor.sh -k ${DTIOUTDIR}/mc_raw_diffusion.nii.
           gz -b raw_diffusion_bval.txt -r ${DTIOUTDIR}/bvec_mc.txt -M
           native_brain_mask.nii.gz -o ${DTIOUTDIR}

#> Calculate RD image from eigenvalues from DTI processing. Scales image to be
   consistent with MD
${DTIOUTDIR}/dti_RD.nii.gz: ${DTIOUTDIR}/dti_FA.nii.gz
        fslmaths ${DTIOUTDIR}/dti_L2.nii.gz -add ${DTIOUTDIR}/dti_L3.nii.gz -div 2
            -mul 1e6 ${DTIOUTDIR}/dti_RD.nii.gz

#> Calculate AD image from eigenvalues from DTI processing. Scales image to be
   consistent with MD
${DTIOUTDIR}/dti_AD.nii.gz: ${DTIOUTDIR}/dti_FA.nii.gz
        fslmaths ${DTIOUTDIR}/dti_L1.nii.gz -mul 1e6 ${DTIOUTDIR}/dti_AD.nii.gz
```

5.2 kurtosis.mk

```
#* DKI preprocessing - takes motion-correctied diffusion data and solves the DKI
   model, using the python package 'dipy'

# PROJECT_HOME should be set elsewhere
# BINDIR=$(PROJECT_HOME)/bin

#! Name the the output directory used for DKI processing
OUTDIR=kurtosis

#! 4mm FWHM (fwhm = 2.3548 * sigma (smoothing factor for kurtosis data))
SIGMA=1.6986

# Set open MP number of threads to be 1, so that we can parallelize using make.
# export OMP_NUM_THREADS=1

.PHONY: kurtosis kurtosis_dir

# keep everything by default
.SECONDARY:

#? Do all kurtosis processing using DKI model with fit_kurtosis.py to generate MK/
   AK/RK maps and using FSL to threshold MK/AK/RK maps from 0-3
kurtosis: kurtosis_dir kurtosis/MK.nii.gz kurtosis/MK_thresh.nii.gz

#? Create the the output directory for kurtosis fitting
kurtosis_dir:
        mkdir -p kurtosis


#> Smooth motion-corrected diffusion data with smoothing factor
kurtosis/sm_mc_raw_diffusion.nii.gz: dti/mc_raw_diffusion.nii.gz
```

```
	fslmaths dti/mc_raw_diffusion.nii.gz -s $(SIGMA) kurtosis/
	   sm_mc_raw_diffusion.nii.gz

#> MK map from DKI processing
kurtosis/MK.nii.gz: kurtosis/sm_mc_raw_diffusion.nii.gz
	$(BINDIR)/fit_kurtosis.py -d kurtosis/sm_mc_raw_diffusion.nii.gz -b
	   raw_diffusion_bval.txt -v dti/bvec_mc.txt -o kurtosis

#> Thresholded MK(0-3)
kurtosis/MK_thresh.nii.gz: kurtosis/MK.nii.gz
	fslmaths kurtosis/MK.nii.gz -thr 0 -min 3 kurtosis/MK_thresh.nii.gz
	fslmaths kurtosis/AK.nii.gz -thr 0 -min 3 kurtosis/AK_thresh.nii.gz
	fslmaths kurtosis/RK.nii.gz -thr 0 -min 3 kurtosis/RK_thresh.nii.gz
```

5.3 kurtosis_unsmoothed.mk

```
#* DKI preprocessing - takes unsmoothed motion-correctied diffusion data and
   solves the dki model, using the python package 'dipy'

# PROJECT_HOME=/mnt/adrc/dki-dbsi
# BINDIR=$(PROJECT_HOME)/bin
# OUTDIR=kurtosis

# 4mm FWHM (fwhm = 2.3548 * sigma)
# SIGMA=1.6986

# Set open MP number of threads to be 1, so that we can parallelize using make.
# export OMP_NUM_THREADS=1

.PHONY: kurtosis-unsmoothed kurtosis_us_dir

# keep everything by default
.SECONDARY:

#? Do all kurtosis processing using DKI model with fit_kurtosis.py to generate
   unsmoothed MK/AK/RK maps and using FSL to threshold unsmoothed MK/AK/RK maps
   from 0-3
kurtosis-unsmoothed: kurtosis_us_dir kurtosis_unsmoothed/MK.nii.gz
   kurtosis_unsmoothed/MK_thresh.nii.gz kurtosis_unsmoothed/MK_thresh_masked.nii.
   gz

#? Create the unsmoothed kurtosis directory
kurtosis_us_dir:
	mkdir -p kurtosis_unsmoothed



#> MK file created from DKI pipeline on unsmoothed data
kurtosis_unsmoothed/MK.nii.gz: dti/mc_raw_diffusion.nii.gz
	$(BINDIR)/fit_kurtosis.py -d dti/mc_raw_diffusion.nii.gz -b
	   raw_diffusion_bval.txt -v dti/bvec_mc.txt -o kurtosis_unsmoothed

#> Thresholded (0-3) measures and generate MK map generated from DKI pipeline in
   unsmoothed data
kurtosis_unsmoothed/MK_thresh.nii.gz: kurtosis_unsmoothed/MK.nii.gz
```

```
        fslmaths kurtosis_unsmoothed/MK.nii.gz -thr 0 -min 3 kurtosis_unsmoothed/
           MK_thresh.nii.gz
        fslmaths kurtosis_unsmoothed/AK.nii.gz -thr 0 -min 3 kurtosis_unsmoothed/
           AK_thresh.nii.gz
        fslmaths kurtosis_unsmoothed/RK.nii.gz -thr 0 -min 3 kurtosis_unsmoothed/
           RK_thresh.nii.gz

#> MK map(0-3) masked out with white matter mask and brain mask in subject
   specific space
kurtosis_unsmoothed/MK_thresh_masked.nii.gz: kurtosis_unsmoothed/MK_thresh.nii.gz
        fslmaths kurtosis_unsmoothed/MK.nii.gz -mas atlas_wm_mask_tosubj.nii.gz -
           mas atlas_brain_mask_tosubj.nii.gz kurtosis_unsmoothed/MK_thresh_masked
           .nii.gz
        fslmaths kurtosis_unsmoothed/AK.nii.gz -mas atlas_wm_mask_tosubj.nii.gz -
           mas atlas_brain_mask_tosubj.nii.gz kurtosis_unsmoothed/AK_thresh_masked
           .nii.gz
        fslmaths kurtosis_unsmoothed/RK.nii.gz -mas atlas_wm_mask_tosubj.nii.gz -
           mas atlas_brain_mask_tosubj.nii.gz kurtosis_unsmoothed/RK_thresh_masked
           .nii.gz
        fslmaths kurtosis_unsmoothed/kurtosis_FA.nii.gz -mas atlas_wm_mask_tosubj.
           nii.gz -mas atlas_brain_mask_tosubj.nii.gz kurtosis_unsmoothed/
           kurtosis_FA_thresh_masked.nii.gz
```

5.4 kurtosis_nob2500.mk

```
#* DKI preprocessing - takes motion-correctied diffusion data with b value no
   greater than 2500 and solves the dki model, using the python package 'dipy'

# PROJECT_HOME should be set elsewhere
# BINDIR=$(PROJECT_HOME)/bin
# OUTDIR=kurtosis

# 4mm FWHM (fwhm = 2.3548 * sigma)
# SIGMA=1.6986

.PHONY: kurtosis kurtosis_dir

# keep everything by default
.SECONDARY:

#? Do all kurtosis processing with b0 values no greater than 2500 using DKI model
   with fit_kurtosis.py to generate MK/AK/RK maps and using FSL to threshold MK/AK
   /RK maps from 0-3
kurtosis: kurtosis_dir kurtosis/MK.nii.gz kurtosis/MK_thresh.nii.gz

#? Create the output directory for kurtosis fitting removing b=2500 images
kurtosis_dir:
        mkdir -p kurtosis


#> Smoothed motion-corrected diffusion data used in DKI pipeline with b0 values no
    greater than 2000
kurtosis/sm_mc_raw_diffusion.nii.gz: dti/mc_raw_diffusion.nii.gz
        fslmaths dti/mc_raw_diffusion.nii.gz -s $(SIGMA) kurtosis/
           sm_mc_raw_diffusion.nii.gz
```

```
#> Remove b=2500 images from smoothing diffusion data
kurtosis/sm_mc_raw_diffusion_nob2500.nii.gz: kurtosis/sm_mc_raw_diffusion.nii.gz
        ../../bin/dti_preproc/rearrange_diffusion.sh kurtosis/sm_mc_raw_diffusion.
           nii.gz raw_diffusion_bval.txt dti/bvec_mc.txt kurtosis/sm_mc_nob2500 1
           2 3 4 5 6 7 8 9 10 11 12 13 14 15 16 17 18 19 20 21 22 23 24 25 26 27
           28 29 30 31 32 33 34 35 36 37 38 39 40 41 42 43 44 45 46 47 48 49 50 51
            52 53 54 55 56 57 58 59 60 61 62 63 64 65 66 67 68 69 70 71 72 73 74
           75 76 77 78 79 80 81 82 83 84 85 86 87 88 89 90 91 92 93 94 95 96 97 98
            99 100 101 102 103 104 105 106 107 108 109 110 111 112 113 114 115 116
            117 118 119 120 121


#>  MK map with b0 values no greater than 2000
kurtosis/MK.nii.gz: kurtosis/sm_mc_raw_diffusion_nob2500.nii.gz
        $(BINDIR)/fit_kurtosis.py -d kurtosis/sm_mc_nob2500_diffusion.nii.gz -b
           kurtosis/sm_mc_nob2500_bvals.txt -v kurtosis/sm_mc_nob2500_bvecs.txt -o
            kurtosis

#> Thresholded MK map(0-3) with b0 value no greater than 2000
kurtosis/MK_thresh.nii.gz: kurtosis/MK.nii.gz
        fslmaths kurtosis/MK.nii.gz -thr 0 -min 3 kurtosis/MK_thresh.nii.gz
        fslmaths kurtosis/AK.nii.gz -thr 0 -min 3 kurtosis/AK_thresh.nii.gz
        fslmaths kurtosis/RK.nii.gz -thr 0 -min 3 kurtosis/RK_thresh.nii.gz
```

5.5 probability_high_kurtosis.mk

```
#* Compute the probablity of white matter in regions of high MK
# ANTs(Advanced Normalization Tools) computes high-dimensional mappings to capture
     the statistics of brain structure and function.
# ANTSpath=/usr/local/ANTs-2.1.0-rc3/bin
# standard FA image
#STDBRAIN=/usr/share/fsl/data/standard/FMRIB58_FA_1mm.nii.gz
#! Standard T1 white matter image
WMATLAS=/mnt/adrc/dki-dbsi/atlas/avg152T1_white.nii.gz

.PHONY: kurtosis_probability probab_dir

#? Make a high MK mask(mask out with whiter matter mask in subject specific space
   and brain mask in subject specific space with threshold of MK>1) and use ANTs
   to register high MK mask to standard space; mask out white matter prior image
   in standard space with the high MK mask in standard space
kurtosis_probability: probab_dir kurtosis_probability/high_MK_mask.nii.gz
   kurtosis_probability/high_MK_mask_tostd.nii.gz kurtosis_probability/
   avg_wm_high_MK_masked.nii.gz

#? Make a directory that will contain high MK mask, high MK mask in standard space
   , white prior in standard space masking out by high MK mask in standrad space
probab_dir:
        mkdir -p kurtosis_probability


#> High MK mask using FSL to mask out MK with whiter matter mask in subject
   specific space and brain mask in subject specific space with threshold of MK>1
kurtosis_probability/high_MK_mask.nii.gz:  kurtosis/MK_thresh.nii.gz
   atlas_wm_mask_tosubj.nii.gz atlas_brain_mask_tosubj.nii.gz
```

```
        fslmaths kurtosis/MK_thresh.nii.gz -mas atlas_wm_mask_tosubj.nii.gz -mas
            atlas_brain_mask_tosubj.nii.gz -thr 1 kurtosis_probability/high_MK_mask
            .nii.gz

#> High MK mask registered to standard FA 1mm image using ants and upscaled into 2
    mm with flirt to be consistent with white prior image in standard space
kurtosis_probability/high_MK_mask_tostd.nii.gz:kurtosis_probability/high_MK_mask.
    nii.gz standard-space/FAtostandard1Warp.nii.gz standard-space/
    FAtostandard0GenericAffine.mat
        antsApplyTransforms -d 3 -i kurtosis_probability/high_MK_mask.nii.gz -o
            kurtosis_probability/high_MK_mask_tostd.nii.gz -t standard-space/
            FAtostandard1Warp.nii.gz -t standard-space/FAtostandard0GenericAffine.
            mat -r ${STDBRAIN};\
        flirt -in kurtosis_probability/high_MK_mask_tostd.nii.gz -ref ${WMATLAS} -
            applyisoxfm 2 -out kurtosis_probability/high_MK_mask_tostd.nii.gz

#> White prior image in standard space masked out with high MK mask in standard
    space using FSL
kurtosis_probability/avg_wm_high_MK_masked.nii.gz:kurtosis_probability/
    high_MK_mask_tostd.nii.gz
        fslmaths ${WMATLAS} -mas kurtosis_probability/high_MK_mask_tostd.nii.gz
            kurtosis_probability/avg_wm_high_MK_masked.nii.gz
```

5.6 registration.mk

```
#* Using ANTs(Advanced Normalization Tools) to register DTI-FA, DBSI-FA and MK to
    standard space, transform white matter mask and brain mask in standard space
    into subject specific space

#! The location of ANTs(Advanced Normalization Tools).
ANTSpath=/usr/local/ANTs-2.1.0-rc3/bin

#! Standard FA image in 1mm space
STDBRAIN=/usr/share/fsl/data/standard/FMRIB58_FA_1mm.nii.gz

.PHONY: register-all reg-dir transform

#? Erode the FA map a little for good registration and register dti_FA/MK/DBSI-FA
    to standard space
register-all: reg-dir  dti/dti_FA_ero.nii.gz standard-space/FAtostandardWarped.nii
    .gz standard-space/MK_tostd.nii.gz dbsi.reorient/raw_dti_fa_map_ss.nii.gz
    standard-space/dbsi_fa_tostd.nii.gz

#? Make a directory for images registered to standard space
reg-dir:
        mkdir -p standard-space

#? Using ANTs to transform white matter mask and brain mask from standard space
    into subject specific space to mask out images in subject specific space
transform: atlas_brain_mask_tosubj.nii.gz  atlas_wm_mask_tosubj.nii.gz

#>  Eroded DTI FA map a little with FSL for good registration
dti/dti_FA_ero.nii.gz: dti/dti_FA.nii.gz
        fslmaths dti/dti_FA.nii.gz -ero -ero dti/dti_FA_ero.nii.gz

#> DTI FA map in standard space using ANTs
```

```
standard-space/FAtostandardWarped.nii.gz: dti/dti_FA_ero.nii.gz
        antsRegistrationSyN.sh -d 3 -f ${STDBRAIN} -m dti/dti_FA_ero.nii.gz -o
           standard-space/FAtostandard -t s

#> DKI MK map in standard space using ANTs
standard-space/MK_tostd.nii.gz: kurtosis/MK_thresh.nii.gz standard-space/
   FAtostandardWarped.nii.gz
        antsApplyTransforms -d 3 -e 3 -i kurtosis/MK_thresh.nii.gz -o standard-
           space/MK_tostd.nii.gz -t standard-space/FAtostandard1Warp.nii.gz -t
           standard-space/FAtostandard0GenericAffine.mat -r ${STDBRAIN}

#> DKI AK map in stardard space using ANTs
standard-space/AK_tostd.nii.gz: kurtosis/AK_thresh.nii.gz standard-space/
   FAtostandardWarped.nii.gz
        antsApplyTransforms -d 3 -e 3 -i kurtosis/AK_thresh.nii.gz -o standard-
           space/AK_tostd.nii.gz -t standard-space/FAtostandard1Warp.nii.gz -t
           standard-space/FAtostandard0GenericAffine.mat -r ${STDBRAIN}

#> Masked out DBSI FA map with brain mask
#  bet b0 iamge native_brain_mask.nii.gz -f (fractional intensity threshold, it
   varies slightly between different subject) for each subject
dbsi.reorient/raw_dti_fa_map_ss.nii.gz: dbsi.reorient/raw_dti_fa_map.nii.gz
   native_brain_mask.nii.gz
        fslmaths dbsi.reorient/raw_dti_fa_map.nii.gz -mas native_brain_mask.nii.gz
            dbsi.reorient/raw_dti_fa_map_ss.nii.gz


#> Eroded DBSI FA map a little with FSL for good registration
 dbsi.reorient/raw_dti_fa_map_ero.nii.gz: dbsi.reorient/raw_dti_fa_map_ss.nii.gz
        fslmaths dbsi.reorient/raw_dti_fa_map_ss.nii.gz -ero -ero  dbsi.reorient/
           raw_dti_fa_map_ero.nii.gz

#> Eroded DBSI FA map in standard space using ANTs
standard-space/dbsi_fa_tostd.nii.gz: standard-space/FAtostandardWarped.nii.gz dbsi
   .reorient/raw_dti_fa_map_ero.nii.gz
        antsApplyTransforms -d 3 -i dbsi.reorient/raw_dti_fa_map_ero.nii.gz -o
           standard-space/dbsi_fa_tostd.nii.gz -t standard-space/FAtostandard1Warp
           .nii.gz -t standard-space/FAtostandard0GenericAffine.mat -r ${STDBRAIN}

#> Brain image in subject specific space using ANTs
atlas_brain_mask_tosubj.nii.gz: dti/dti_FA_ero.nii.gz standard-space/
   FAtostandard1InverseWarp.nii.gz standard-space/FAtostandard0GenericAffine.mat
        antsApplyTransforms -d 3 -r dti/dti_FA_ero.nii.gz -t standard-space/
           FAtostandard1InverseWarp.nii.gz -t [standard-space/
           FAtostandard0GenericAffine.mat,1] -n NearestNeighbor -i ${STDBRAIN} -o
           atlas_brain_mask_tosubj.nii.gz

#> White matter mask in subject specific space using ANTs
atlas_wm_mask_tosubj.nii.gz: dti/dti_FA_ero.nii.gz standard-space/
   FAtostandard1InverseWarp.nii.gz standard-space/FAtostandard0GenericAffine.mat
        antsApplyTransforms -d 3 -r dti/dti_FA_ero.nii.gz -t standard-space/
           FAtostandard1InverseWarp.nii.gz -t [standard-space/
           FAtostandard0GenericAffine.mat,1] -n NearestNeighbor -i ../../atlas/
           white_matter_mask.nii.gz -o atlas_wm_mask_tosubj.nii.gz
```

5.7 reorientation_dbsi.mk

```
#* Reorder the data storage to permit changes between axial,sagital and coronal
   slicing to change the orientation information in the header as well as
   reorerding the data: delete the orientation information and reset the
   orientation information by copying the geometric information form DTI FA maps

reorientedfiles:= $(subst dbsi,dbsi.reorient, $(wildcard dbsi/*.nii))
compressedfiles:= $(subst .nii,.nii.gz,$(reorientedfiles))

#? Reorient the DBSI raw data obtained from Dr.Wang to FSL orientation
reorient-all: $(compressedfiles)


#> Reoriented DBSI raw data obtained from Dr.Wang
dbsi.reorient/%.nii.gz : dbsi/%.nii dti/dti_FA_ero.nii.gz
        mkdir -p dbsi.reorient ;\
        fslswapdim dbsi/$*.nii -x -y z $@          &&\
        fslcpgeom dti/dti_FA_ero.nii.gz $@
```

5.8 dbsi_mask.mk

```
#* Use FSL to mask out DBSI water(WR), hindered(HR) and restricted(RR) ratio maps
   with high MK mask and compute the maximum of water, hindered and restricted
   ratio maps for each individual; Use ANTs to register fiber, water, hindered and
    restricted maps and WR,HR and RR masked out by high MK mask to standard FA map
    in 1mm space

# standard FA image
# STDBRAIN=/usr/share/fsl/data/standard/FMRIB58_FA_1mm.nii.gz

.PHONY:dbsi.mask-dir mk_mask-all dbsi.mask-all register_tostd ratio_masked_max

#? Make the high(>1) MK mask masking out with white matter mask in subject
   specific space
mk_mask-all: MK_thresh_mask.nii.gz high_MK_mask.nii.gz

#? Mask out DBSI-FR/RR/WR/HR with high MK mask(>1)
dbsi.mask-all: dbsi.mask-dir dbsi.mask/fiber_ratio_masked.nii.gz dbsi.mask/
   restricted_ratio_masked.nii.gz dbsi.mask/water_ratio_masked.nii.gz dbsi.mask/
   hindered_ratio_masked.nii.gz

#? Make a directory that will contain masked out DBSI FR/RR/WR/HR map
dbsi.mask-dir:
        mkdir -p dbsi.mask

#? Use ANTs to register DBSI FR/RR/WR/HR map to standard space and the masked DBSI
   -FR/RR/WR/HR to standard space
register_tostd:dbsi.mask/water_ratio_masked_tostd.nii.gz dbsi.mask/
   hindered_ratio_masked_tostd.nii.gz dbsi.mask/restricted_ratio_masked_tostd.nii.
   gz dbsi.mask/raw_restricted_ratio_map_tostd.nii.gz dbsi.mask/
   raw_hindered_ratio_map_tostd.nii.gz dbsi.mask/raw_water_ratio_map_tostd.nii.gz
   dbsi.mask/raw_fiber_ratio_map_tostd.nii.gz

#? Use FSL to make the FR/HR/RR/WR maximum ratio image
ratio_masked_max: dbsi.mask/max_output.nii.gz dbsi.mask/hindered_ratio_masked_max.
   nii.gz dbsi.mask/restricted_ratio_masked_max.nii.gz dbsi.mask/
   water_ratio_masked_max.nii.gz
```

```
#> MK mask(0-3) masked out with white matter mask in subject specific space
MK_thresh_mask.nii.gz: kurtosis/MK_thresh.nii.gz atlas_wm_mask_tosubj.nii.gz
        fslmaths kurtosis/MK_thresh.nii.gz -mas atlas_wm_mask_tosubj.nii.gz
           MK_thresh_mask.nii.gz

#> High MK mask(>1) in subject specific space using FSL
high_MK_mask.nii.gz: MK_thresh_mask.nii.gz
        fslmaths MK_thresh_mask.nii.gz -thr 1 -bin high_MK_mask.nii.gz

#> DBSI fiber ratio map masked out with high MK mask(>1) using FSL
dbsi.mask/fiber_ratio_masked.nii.gz: dbsi.reorient/raw_fiber_ratio_map.nii.gz
   high_MK_mask.nii.gz
        fslmaths dbsi.reorient/raw_fiber_ratio_map.nii.gz -mas high_MK_mask.nii.gz
            dbsi.mask/fiber_ratio_masked.nii.gz

#> DBSI restricted ratio map masked out with high MK mask (>1) using FSL
dbsi.mask/restricted_ratio_masked.nii.gz:  dbsi.reorient/raw_restricted_ratio_map.
   nii.gz high_MK_mask.nii.gz
        fslmaths dbsi.reorient/raw_restricted_ratio_map.nii.gz -mas high_MK_mask.
           nii.gz dbsi.mask/restricted_ratio_masked.nii.gz

#> DBSI water ratio map masked out with high MK mask(>1)using FSL
dbsi.mask/water_ratio_masked.nii.gz: dbsi.reorient/raw_water_ratio_map.nii.gz
   high_MK_mask.nii.gz
        fslmaths dbsi.reorient/raw_water_ratio_map.nii.gz -mas high_MK_mask.nii.gz
            dbsi.mask/water_ratio_masked.nii.gz

#> DBSI hindered ratio map masked out with high MK mask(>1)using FSL
dbsi.mask/hindered_ratio_masked.nii.gz: dbsi.reorient/raw_hindered_ratio_map.nii.
   gz high_MK_mask.nii.gz
        fslmaths dbsi.reorient/raw_hindered_ratio_map.nii.gz -mas high_MK_mask.nii
           .gz dbsi.mask/hindered_ratio_masked.nii.gz


#>  Masked(MK>1) DBSI water ratio map in standard FA image in 1mm space using ANTs
dbsi.mask/water_ratio_masked_tostd.nii.gz: dbsi.mask/water_ratio_masked.nii.gz
   standard-space/FAtostandardWarped.nii.gz standard-space/
   FAtostandard0GenericAffine.mat
        antsApplyTransforms -d 3 -i dbsi.mask/water_ratio_masked.nii.gz -o dbsi.
           mask/water_ratio_masked_tostd.nii.gz -r ${STDBRAIN} -t standard-space/
           FAtostandardWarped.nii.gz -t standard-space/FAtostandard0GenericAffine.
           mat

#> Masked(MK>1) DBSI restricted ratio map in standard FA image in 1mm space using
   ANTs
dbsi.mask/restricted_ratio_masked_tostd.nii.gz: dbsi.mask/restricted_ratio_masked.
   nii.gz standard-space/FAtostandardWarped.nii.gz standard-space/
   FAtostandard0GenericAffine.mat
        antsApplyTransforms -d 3 -i dbsi.mask/restricted_ratio_masked.nii.gz -o
           dbsi.mask/restricted_ratio_masked_tostd.nii.gz -r ${STDBRAIN} -t
           standard-space/FAtostandardWarped.nii.gz -t standard-space/
           FAtostandard0GenericAffine.mat
```

```
#> Masked(MK>1) DBSI hindered ratio map in standard FA image in 1mm space using
   ANTs
dbsi.mask/hindered_ratio_masked_tostd.nii.gz: dbsi.mask/hindered_ratio_masked.nii.
   gz standard-space/FAtostandardWarped.nii.gz standard-space/
   FAtostandard0GenericAffine.mat
	antsApplyTransforms -d 3 -i dbsi.mask/hindered_ratio_masked.nii.gz -o dbsi
	   .mask/hindered_ratio_masked_tostd.nii.gz -r ${STDBRAIN} -t standard-
	   space/FAtostandardWarped.nii.gz -t standard-space/
	   FAtostandard0GenericAffine.mat

#> Maximum ratio of masked(MK>1) water,restricted and hindered ratios
 dbsi.mask/max_output.nii.gz:  dbsi.mask/hindered_ratio_masked.nii.gz dbsi.mask/
    water_ratio_masked.nii.gz dbsi.mask/restricted_ratio_masked.nii.gz
	fslmaths dbsi.mask/hindered_ratio_masked.nii.gz -max dbsi.mask/
	   water_ratio_masked.nii.gz -max dbsi.mask/restricted_ratio_masked.nii.gz
	    dbsi.mask/max_output.nii.gz

#> Maximum ratio of masked(MK>1)hindered ratio map
dbsi.mask/hindered_ratio_masked_max.nii.gz: dbsi.mask/hindered_ratio_masked.nii.gz
    dbsi.mask/max_output.nii.gz
	fslmaths dbsi.mask/hindered_ratio_masked.nii.gz -sub dbsi.mask/max_output.
	   nii.gz -add 1 -thr 1 -mul dbsi.mask/hindered_ratio_masked.nii.gz dbsi.
	   mask/hindered_ratio_masked_max.nii.gz

#> Maximum ratio of masked(MK>1)restricted ratio map
dbsi.mask/restricted_ratio_masked_max.nii.gz: dbsi.mask/restricted_ratio_masked.
   nii.gz dbsi.mask/max_output.nii.gz
	fslmaths dbsi.mask/restricted_ratio_masked.nii.gz -sub dbsi.mask/
	   max_output.nii.gz -add 1 -thr 1 -mul dbsi.mask/restricted_ratio_masked.
	   nii.gz dbsi.mask/restricted_ratio_masked_max.nii.gz

#> Maximum ratio of masked(MK>1)water ratio map
dbsi.mask/water_ratio_masked_max.nii.gz: dbsi.mask/water_ratio_masked.nii.gz dbsi.
   mask/max_output.nii.gz
	fslmaths dbsi.mask/water_ratio_masked.nii.gz -sub dbsi.mask/max_output.nii
	   .gz -add 1 -thr 1 -mul dbsi.mask/water_ratio_masked.nii.gz dbsi.mask/
	   water_ratio_masked_max.nii.gz

#> DBSI fiber ratio map in standard space using ANTs
dbsi.mask/raw_fiber_ratio_map_tostd.nii.gz:dbsi.reorient/raw_fiber_ratio_map.nii.
   gz
	antsApplyTransforms -d 3 -i dbsi.reorient/raw_fiber_ratio_map.nii.gz -o
	   dbsi.mask/raw_fiber_ratio_map_tostd.nii.gz -t standard-space/
	   FAtostandard1Warp.nii.gz -t standard-space/FAtostandard0GenericAffine.
	   mat -r ${STDBRAIN}

#> DBSI water ratio map in standard space using ANTs
dbsi.mask/raw_water_ratio_map_tostd.nii.gz:dbsi.reorient/raw_water_ratio_map.nii.
   gz
	antsApplyTransforms -d 3 -i dbsi.reorient/raw_water_ratio_map.nii.gz -o
	   dbsi.mask/raw_water_ratio_map_tostd.nii.gz -t standard-space/
	   FAtostandard1Warp.nii.gz -t standard-space/FAtostandard0GenericAffine.
	   mat -r ${STDBRAIN}

#> DBSI hindered ratio map in standard space using ANTs
```

```
dbsi.mask/raw_hindered_ratio_map_tostd.nii.gz: dbsi.reorient/
   raw_hindered_ratio_map.nii.gz
        antsApplyTransforms -d 3 -i dbsi.reorient/raw_hindered_ratio_map.nii.gz -o
            dbsi.mask/raw_hindered_ratio_map_tostd.nii.gz -t standard-space/
           FAtostandard1Warp.nii.gz -t standard-space/FAtostandard0GenericAffine.
           mat -r ${STDBRAIN}

#> DBSI restricted ratio map in standard space using ANTs
dbsi.mask/raw_restricted_ratio_map_tostd.nii.gz:dbsi.reorient/
   raw_restricted_ratio_map.nii.gz
        antsApplyTransforms -d 3 -i dbsi.reorient/raw_restricted_ratio_map.nii.gz
           -o dbsi.mask/raw_restricted_ratio_map_tostd.nii.gz -t standard-space/
           FAtostandard1Warp.nii.gz -t standard-space/FAtostandard0GenericAffine.
           mat -r ${STDBRAIN}
```

5.9 correlogram.mk

```
#* Use FSL to mask out DTI, DKI, DBSI parameters that will be used to compute the
   correlation between the three models with white matter mask and brain mask in
   subject specific space

.PHONY:correlogram-dbsi.masked correlogram-fa.masked correlogram-kurtosis.masked
   correlo-dir

#? Use FSL to mask out DBSI ratio map with white matter mask and brain mask in
   subject specific space
correlogram-dbsi.masked: correlo-dir correlogram/fiber_ratio_map_masked.nii.gz
   correlogram/hindered_ratio_map_masked.nii.gz correlogram/
   restricted_ratio_map_masked.nii.gz correlogram/water_ratio_map_masked.nii.gz

#? Mask out DTI/DKI/DBSI FA maps with white matter mask and brain mask in subject
   specific space
correlogram-fa.masked: correlogram/dti_FA_ero_masked.nii.gz correlogram/
   kurtosis_FA_masked.nii.gz correlogram/dbsi_fa_map_masked.nii.gz

#? Mask out DKI MK/RK/AK map with white matter mask and brain mask in subject
   specific space
correlogram-kurtosis.masked: correlogram/MK_thresh_masked.nii.gz correlogram/
   AK_thresh_masked.nii.gz correlogram/RK_thresh_masked.nii.gz correlogram/
   kurtosis_FA_masked.nii.g

#? Make a directory that will contain masked DTI, DKI and DBSI parameters
correlo-dir:
        mkdir -p correlogram

#> DBSI fiber ratio map masked out with white matter mask and brain mask in
   subject specific space using FSL
correlogram/fiber_ratio_map_masked.nii.gz:dbsi.reorient/raw_fiber_ratio_map.nii.gz
        fslmaths dbsi.reorient/raw_fiber_ratio_map.nii.gz -mas
           atlas_wm_mask_tosubj.nii.gz -mas atlas_brain_mask_tosubj.nii.gz
           correlogram/fiber_ratio_map_masked.nii.gz

#> DBSI water ratio map masked out with white matter mask and brain mask in
   subject specific space using FSL
correlogram/water_ratio_map_masked.nii.gz: dbsi.reorient/raw_water_ratio_map.nii.
   gz
```

```
        fslmaths dbsi.reorient/raw_water_ratio_map.nii.gz -mas
           atlas_wm_mask_tosubj.nii.gz -mas atlas_brain_mask_tosubj.nii.gz
           correlogram/water_ratio_map_masked.nii.gz

#> DBSI hindered ratio map masked out with white matter mask and brain mask in
   subject specific space using FSL
correlogram/hindered_ratio_map_masked.nii.gz:dbsi.reorient/raw_hindered_ratio_map.
   nii.gz
        fslmaths dbsi.reorient/raw_hindered_ratio_map.nii.gz -mas
           atlas_wm_mask_tosubj.nii.gz -mas atlas_brain_mask_tosubj.nii.gz
           correlogram/hindered_ratio_map_masked.nii.gz

#> DBSI restricted ratio map masked out with white matter mask and brain mask in
   subject specific space using FSL
correlogram/restricted_ratio_map_masked.nii.gz:dbsi.reorient/
   raw_restricted_ratio_map.nii.gz
        fslmaths dbsi.reorient/raw_restricted_ratio_map.nii.gz -mas
           atlas_wm_mask_tosubj.nii.gz -mas atlas_brain_mask_tosubj.nii.gz
           correlogram/restricted_ratio_map_masked.nii.gz

#> MK(0-3) masked out with whiter matter mask and brain mask in subject specific
   space using FSL
correlogram/MK_thresh_masked.nii.gz: kurtosis/MK_thresh.nii.gz
        fslmaths kurtosis/MK_thresh.nii.gz -mas atlas_wm_mask_tosubj.nii.gz -mas
           atlas_brain_mask_tosubj.nii.gz correlogram/MK_thresh_masked.nii.gz

#> DKI AK map masked out with white matter mask and brain mask in subject specific
    space using FSL
correlogram/AK_thresh_masked.nii.gz: kurtosis/AK_thresh.nii.gz
        fslmaths kurtosis/AK_thresh.nii.gz -mas atlas_wm_mask_tosubj.nii.gz -mas
           atlas_brain_mask_tosubj.nii.gz correlogram/AK_thresh_masked.nii.gz

#> DKI RK map masked out with white matter mask and brain mask in subject specific
    space using FSL
correlogram/RK_thresh_masked.nii.gz: kurtosis/RK_thresh.nii.gz
        fslmaths kurtosis/RK_thresh.nii.gz -mas atlas_wm_mask_tosubj.nii.gz -mas
           atlas_brain_mask_tosubj.nii.gz correlogram/RK_thresh_masked.nii.gz

#> Eroded DTI FA map masked out with white matter mask and brain mask in subject
   specific space using FSL
correlogram/dti_FA_ero_masked.nii.gz:dti/dti_FA_ero.nii.gz
        fslmaths dti/dti_FA_ero.nii.gz -mas atlas_wm_mask_tosubj.nii.gz -mas
           atlas_brain_mask_tosubj.nii.gz correlogram/dti_FA_ero_masked.nii.gz

#> DKI FA map masked out with white matter mask and brain mask in subject specific
    space using FSL
 correlogram/kurtosis_FA_masked.nii.gz:kurtosis/kurtosis_FA.nii.gz
        fslmaths kurtosis/kurtosis_FA.nii.gz -mas atlas_wm_mask_tosubj.nii.gz -mas
            atlas_brain_mask_tosubj.nii.gz correlogram/kurtosis_FA_masked.nii.gz

#> DBSI FA map masked out with white matter mask and brain mask in subject
   specific space using FSL
 correlogram/dbsi_fa_map_masked.nii.gz:dbsi.reorient/raw_dti_fa_map_ero.nii.gz
        fslmaths dbsi.reorient/raw_dti_fa_map_ero.nii.gz -mas atlas_wm_mask_tosubj
           .nii.gz -mas atlas_brain_mask_tosubj.nii.gz correlogram/
           dbsi_fa_map_masked.nii.gz
```
